# High-efficiency, flexible and large-area red/green/blue all-inorganic metal halide perovskite quantum wires-based light-emitting diodes

**DOI:** 10.1038/s41467-023-40150-y

**Published:** 2023-08-01

**Authors:** Yang Bryan Cao, Daquan Zhang, Qianpeng Zhang, Xiao Qiu, Yu Zhou, Swapnadeep Poddar, Yu Fu, Yudong Zhu, Jin-Feng Liao, Lei Shu, Beitao Ren, Yucheng Ding, Bing Han, Zhubing He, Dai-Bin Kuang, Kefan Wang, Haibo Zeng, Zhiyong Fan

**Affiliations:** 1 Department of Electronic & Computer Engineering, The Hong Kong University of Science and Technology, Clear Water Bay, Kowloon, Hong Kong SAR China; 2 State Key Laboratory of Advanced Display and Optoelectronics Technologies HKUST, Clear Water Bay, Kowloon, Hong Kong SAR China; 3 Guangdong-Hong Kong-Macau Joint Laboratory for Intelligent Micro-Nano Optoelectronic Technology, HKUST, Clear Water Bay, Kowloon, Hong Kong SAR China; 4 Department of Materials Science and Engineering, Southern University of Science and Technology, No. 1088, Xueyuan Rd., Shenzhen, 518055 Guangdong China; 5 MOE Key Laboratory of Bioinorganic and Synthetic Chemistry, Lehn Institute of Functional Materials, School of Chemistry, Sun Yat‐sen University, Guangzhou, 510275 China; 6 Henan Provinces Key Laboratory of Photovoltaic Materials, Henan University, Kaifeng, 475004 Henan China; 7 MIIT Key Laboratory of Advanced Display Materials and Devices, Institute of Optoelectronics & Nanomaterials, School of Materials Science and Engineering, Nanjing University of Science and Technology, Nanjing, 210094 China

**Keywords:** Inorganic LEDs, Nanowires

## Abstract

Metal halide perovskites have shown great promise as a potential candidate for next-generation solid state lighting and display technologies. However, a generic organic ligand-free and antisolvent-free solution method to fabricate highly efficient full-color perovskite light-emitting diodes has not been realized. Herein, by utilizing porous alumina membranes with ultra-small pore size as templates, we have successfully fabricated crystalline all-inorganic perovskite quantum wire arrays with ultrahigh density and excellent uniformity, using a generic organic ligand-free and anti-solvent-free solution method. The quantum confinement effect, in conjunction with the high light out-coupling efficiency, results in high photoluminescence quantum yield for blue, sky-blue, green and pure-red perovskite quantum wires arrays. Consequently, blue, sky-blue, green and pure-red LED devices with spectrally stable electroluminescence have been successfully fabricated, demonstrating external quantum efficiencies of 12.41%, 16.49%, 26.09% and 9.97%, respectively, after introducing a dual-functional small molecule, which serves as surface passivation and hole transporting layer, and a halide vacancy healing agent.

## Abstract (web summary)

Cao et al. reports highly efficient, flexible, and larger-area Red/Green/Blue perovskite light-emitting diodes based on all-inorganic metal halide perovskite quantum wires arrays fabricated by porous alumina membranes assisted solution method.

## Introduction

Metal halide perovskites (MHPs) have proven to be propitious candidates for next-generation solid-state lighting and display technology owing to their excellent properties such as high photoluminescence quantum yield (PLQY), tunable emission wavelength, narrow emission linewidth, low trap-state density, high charge carrier mobility, solution feasibility and easy-avaliability^1–5^. Rapid development has been made to improve the external quantum efficiencies (EQEs) of PeLEDs from less than 1% to over 20% within just a decade, since the first report of room temperature electroluminescence (EL) in 2014^6–10^. Despite the significant advancements achieved on green, red and near infrared perovskite light-emitting diodes (PeLEDs), the best reported EQE of blue PeLEDs is merely 18%^11^, which impedes their practical applications in wide-color-gamut displays and solid-state lighting. Therefore, marked efforts are desired to develop a universal fabrication method for simultaneously achieving highly efficient blue, green and red PeLEDs.

In fact, full-color light emission from MHPs can be readily realized by introducing quantum confinement effect^12,13^ or engineering halide composition^2^. However, according to a theoretical study of CsPbX_3_ nanocrystal system^14^, halide vacancies induced local point defects can easily accumulate at the crystal surface, resulting in obvious reduction of their PLQY^15^. Meanwhile, increasing halide dopant in mixed-halide perovskites deteriorates the morphology, resulting in non-uniform light emission and unfavorable carrier injection^16,17^. Moreover, the EL spectrum instability is a severe issue for the mixed-halide PeLEDs, which can be ascribed to the phase segregation and ion migration under electrical field^18^.

In order to address these challenging issues above, organic ligands are commonly used to form quasi-2D perovskites with high PLQY and good film morphology^16,19–21^, subsequently leading to high EQEs. Nevertheless, the coexistence of variable *n* phases might contribute to high nonradiative recombination and broadened light emission without careful design of the distribution of different phases^19,22^. Besides, the aggregated organic ligands can be decomposed because of the internal Joule heat during operation^10^. Antisolvent treatment has also become the mainstream technique to further improve the film quality, yielding high-performance PeLEDs^16,21,23,24^. However, the time, volume and position of the antisolvent dropping need to be precisely controlled to avoid detrimental impact on the film quality. Furthermore, the commonly used antisolvents such as chlorobenzene and chloroform are environmentally unfriendly and highly toxic to human body. Even though decent improvements have been made through introducing organic ligands and engineering antisolvent treatment^16,20,23,25–27^, organic ligand-free and antisolvent-free approach has rarely been explored with the similar improvement in the PeLEDs performance.

Recently, we demonstrated that porous alumina membranes (PAMs) can be used as excellent templates to guide vapor phase growth of PeQWs and fabricated large area green PeLEDs with unique shapes^28^. Here we employ PAMs with ultra-small pore size (~6.4 nm diameter) to assist the formation of crystalline rubidium bromide (RbBr) doped all-inorganic perovskite (Rb: CsPbX_3_, X = Cl, Br, I) quantum wires (PeQWs) through organic ligand-free and antisolvent-free solution method. Benefiting from the quantum confinement effect, high light out-coupling efficiency (OCE) as well as the surface passivation effect from the PAM template, 24%, 73%, 92% and 55% PLQYs have been achieved for blue, sky-blue, green and pure-red PeQWs, respectively. Intriguingly, the incorporation of small amount of RbBr suppresses the nonradiative recombination and halide segregation in PeQWs. With a dual-functional small molecule, 1,1-Bis[(di-4-tolylamino)phenyl]cyclohexane (TAPC) which serves as passivation layer and hole transporting layer, and a surface-stabilizing 1,3,5-tris(bromomethyl)−2,4,6-triethylbenzene (TBTB), blue, sky-blue, green and pure-red PeLEDs are successfully fabricated with maximum EQEs of 12.41%, 16.49%, 26.09% and 9.97%, respectively. Note that our devices are top-emission devices, and here we report the highest efficiency top-emission blue PeLED so far. To our best knowledge, the 16.49% EQE for sky-blue color is the record for all-inorganic sky-blue PeLEDs. The 26.09% EQE for green LED is the record for all-inorganic PeLEDs and it is also the record for all top-emission PeLEDs so far. More importantly, there is no discernible EL peak wavelength drifting observed during the device operation. Finally, uniform light emission from flexible (1.5 × 1.5 cm^2^) and large-area (3 × 3.5 cm^2^) devices were successfully illustrated. As such, the unique and generic strategy developed here, leveraging quantum confinement and template packaging of perovskite materials, has demonstrated its promising potency to achieve high performance and spectrally stable PeLEDs for future full-color displays and solid-state lighting applications.

## Results

**Fig. 1:**
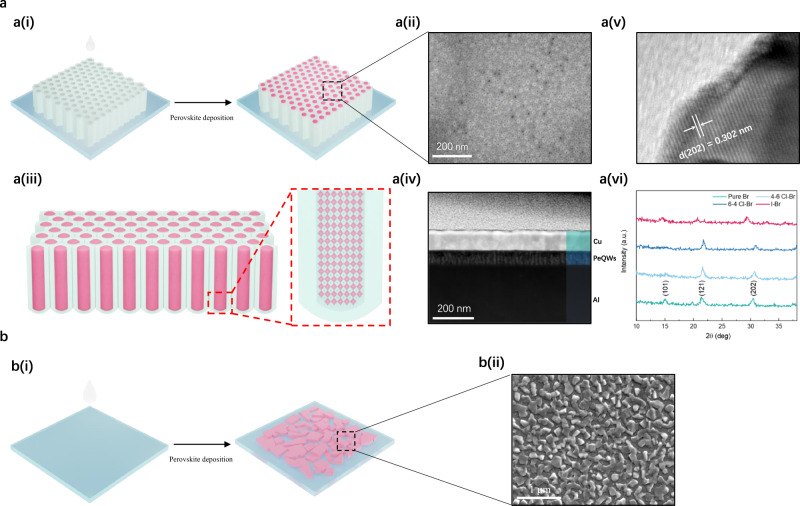
Deposition and formation of PeQW arrays. **a** **(i)** Schematic illustrations of the perovskite deposition process on PAM template. **(ii)** Top view SEM images of the PeQWs. **(iii)** Schematic drawing of cross-sectional view of perovskite QWs arrays. **(iv)** The cross-sectional TEM image of PeQW arrays. **(v)** HRTEM image of CsPbBr_3_ QW extracted from PAM template. **(vi)** XRD patterns of the four different PeQW arrays in PAM. **b** **(i)** Schematic illustrations of the perovskite deposition process on ITO glass. **(ii)** Top view SEM images of the thin film.

The perovskite precursor solution is prepared by dissolving the mixture of RbBr, CsBr, PbBr_2_ and PbX_2_ (X = Cl or I) in DMSO with different molar ratios. Incorporating Rb^+^ ions in perovskites aims at suppressing non-radiation recombination and halide segregation, which has been reported elsewhere^29,30^. Typically, four precursors with different molar ratios, namely RbBr:CsBr:PbBr_2_:PbCl_2_ = 0.1:1.4:0.4:0.6 (6-4 Cl–Br sample), 0.1:1.4:0.6:0.4 (4–6 Cl-Br sample), 0.1:1.4:1:0 (pure Br sample) and RbBr:CsBr:PbI_2_ = 0.1:1.2:1 (I–Br sample), are prepared and studied, corresponding to the blue, sky-blue, green and pure-red emitting PeQWs, respectively. The purpose of adding excess CsBr for all precursor solutions is to eliminate non-perovskite phase, eventually achieving higher device performance^25,31,32^. The fabrication processes of PeQWs and perovskite thin film are schematically illustrated in Fig. 1a(i), b(i), showing a conventional spin-coating method to deposit perovskite on two different substrates. As shown in Fig. 1a(i), once the perovskite precursor solution drops onto the PAMs, precursor solution will infuse into the channels of PAMs followed by a spinning process for residual solution removal and a post annealing process for crystallization. As for the control sample, the same precursor solution is spin-coated on a planar ITO glass for comparison (Fig. 1b(i)). It is noted that no organic ligand or antisolvent is used in spin-coating processes. The scanning electron microscopy (SEM) image in Fig. 1b(ii) reveals that a discontinuous CsPbBr_3_ film with excessive amount of pin holes is obtained on blank ITO glass without applying ligand and antisolvent. Meanwhile, the increase in halide dopants concentration makes the perovskite thin film morphology even worse (Supplementary Fig. 1a–d), which is in congruence with previous reports^16,33^. In stark contrast, highly uniform PeQW arrays with high filling density embedded in PAMs with an average pore diameter of 6.4 nm (Supplementary Fig. 2a) are acquired, as shown in Fig. 1a(ii). Although few empty holes are observed, it doesn’t have detrimental effect on the performance of PeLEDs due to the insulating nature of aluminum oxide. In addition, no obvious change on the morphologies is observed after halide doping (Supplementary Fig. 2b–e). The cross-sectional transmission electron microscopy (TEM) image and high-resolution TEM (HRTEM) images are collected to confirm the existence of PeQWs (Fig. 1a(iii), (iv), (v)). HRTEM image in Fig. 1a(v) shows the interplane spacing of CsPbBr_3_ QW is 3.02 Å which can be assigned to the distance between two neighboring (202) planes. HRTEM images of single PeQWs can be found in Supplementary Fig. 3. X-ray diffraction (XRD) patterns are measured to study the crystal structure of PeQWs which are shown in Fig. 1a(vi). The patterns show distinctive peaks at ~15.13°, ~21.43° and ~30.43° which can be classified to (101), (121) and (202) planes for orthorhombic CsPbBr_3_ phase, respectively. Meanwhile, introducing smaller chlorine atoms into CsPbBr_3_ lattice decreases the lattice constant, leading to a peak position shift to a larger angle. On the contrary, incorporating iodine atoms into CsPbBr_3_ lattice shows an opposite tendency.

**Fig. 2:**
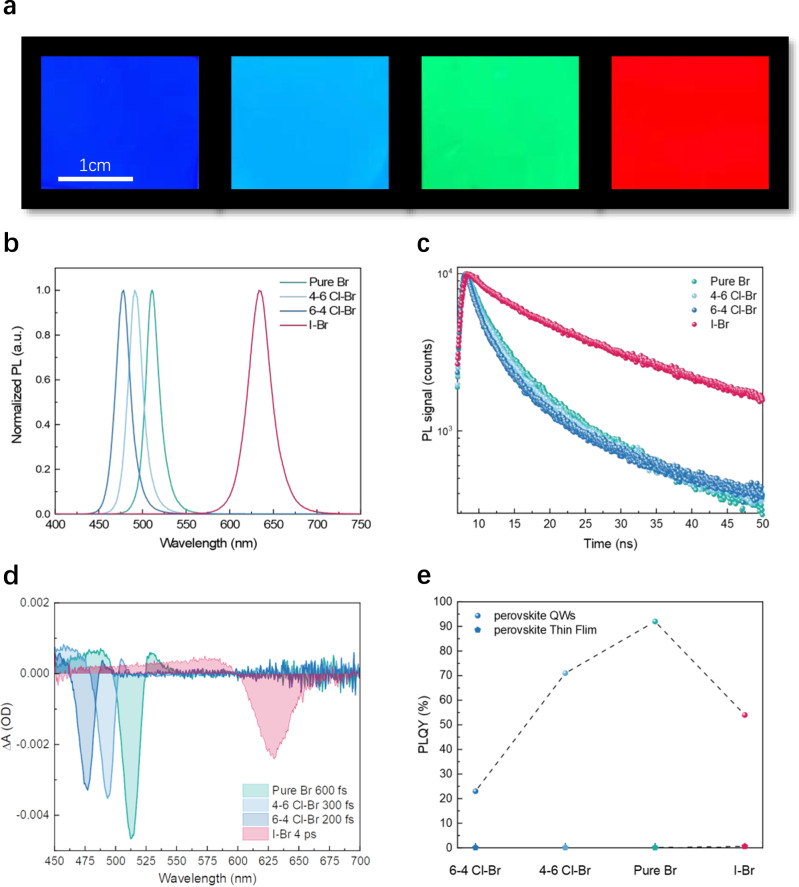
Optical properties of PeQWs. **a** Photograph of blue, sky-blue, green and pure-red light-emitting PeQW arrays under UV irradiation. **b** Normalized photoluminescence spectra (λ_ex_ = 350 nm) of our PeQW arrays. **c** Time-resolved photoluminescence decay curve (λ_ex_ = 365 nm) measured at corresponding photoluminescence peaks for the corresponding PeQW arrays. **d** TA spectra of four different PeQW arrays after excitation at 405 nm. **e** PLQYs of PeQWs and perovskite thin films.

Combining quantum confinement effect with halide compositional stoichiometry engineering can potentially realize light emissions covering the whole visible range. Bright and uniform photoluminescence (PL) from four different kinds of PeQWs under ultraviolet (UV) irradiation (365 nm) are demonstrated in Fig. 2a. The emission colors of these four kinds of PeQWs are blue, sky-blue, green and pure-red, respectively, covering almost the whole visible range. The photoluminescent mapping analysis in Supplementary Fig. 4 verifies excellent optical homogeneity of the PeQW arrays. The corresponding steady state PL spectra are shown in Fig. 2b, exhibiting peaks at 478 nm (blue), 491 nm (sky-blue), 512 nm (green) and 630 nm (pure-red). Time-resolved photoluminescence (TRPL) measurement is carried out to investigate the excited charge carrier lifetime (Fig. 2c). The PL decay curves (Supplementary Fig. 5) are fitted in two regions, where a short lifetime is related with trap-mediated nonradiative recombination and the longer lifetime can be assigned to radiative recombination^34^. The average carrier lifetime (τ_avg_) of four kinds of PeQWs are 5.45 ns (blue), 6.78 ns (sky-blue), 7.40 ns (green) and 12.94 ns (pure-red), respectively. The reduction in average carrier lifetime with the increase of chloride content can be partially ascribed to the increase of defects induced by halide vacancies, which can be proved by the total hole trap-state density calculated from the *J*–*V* characteristics of hole-only devices (Supplementary Fig. 6). The Urbach energy is also calculated from the absorption coefficient spectrum extracted from the absorbance spectrum (Supplementary Fig. 7). Ultrafast transient absorption (TA) spectroscopy measurement has been conducted to reveal the transfer and recombination dynamics of photogenerated carriers in PeQWs. As shown in Fig. 2d, a single pronounced ground-state bleach (GSB) peak is recorded for four different PeQWs that coincides with the absorption edge (Supplementary Fig. 8). According to the TA spectra in Supplementary Fig. 9, a single relatively narrow GSB peak can be found, indicating that no obvious energy transfer is observed and PeQW is of relatively high compositionally homogenous. Here, the term “compositional homogeneity” indicates the halide anions are uniformly distributed in mixed-halide perovskite. It has been reported that a high compositional homogeneity can significantly improve the phase stability, thus suppressing the halide segregation^29,35^. Without organic ligand engineering and antisolvent treatment, discontinuous films will form for CsPbBr_3_ and Cs-based mixed-halide perovskites with abundant pin holes and high trap density on planar substrates^19,36^. Therefore, the PLQYs of these thin films are typically less than 1% (Fig. 2e). Conversely, PeQWs in PAMs have a much higher PLQY which can be attributed to improved crystal quality, quantum confinement effect^12,37,38^, passivation by aluminum oxide and enhanced light extraction^12^. The high exciton binding energy of PeQWs indicates that perovskite experiences a strong quantum confinement effect from the diameter reduction (Supplementary Figs. 10 and 11). Additionally, the passivation of aluminum oxide also can be confirmed by the X-ray photoelectron spectroscopy (XPS) results which are shown in Supplementary Fig. 12. The Pb 4*f* spectrum for PeQWs exhibits two dominant peaks situated at 138.6 eV and 143.5 eV, associating with 4*f*_7/2_ and 4*f*_5/2_ orbitals. Two Br binding energy peaks, corresponding to 3d_5/2_ (68.5 eV) and 3d_3/2_ (69.3 eV) levels, are observed in the Br 3*d* spectrum. Compared with perovskite thin film, Pb 4*f* peaks shift to a lower binding energy which indicates that the existence of under-coordinated Pb ions and aluminum oxide interaction. Consistent with the reported results and density functional theory calculations (DFT) results done by us^39,40^, such a shift is trigged by the bonding between the under-coordinated Pb ions and the oxygen ions, which can effectively suppress nonradiative recombination on the perovskite surface.

**Fig. 3:**
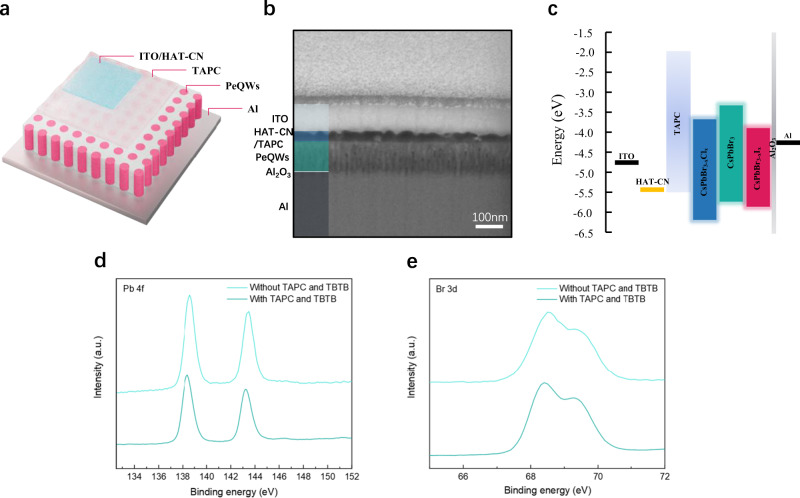
PeQWs-based LED device structure and passivation of TPAC and TBTB. **a** Schematic of the of PeQWs-based LED device structure. **b** An SEM image showing the cross-section of the device **c** Energy band diagram of each functional layer. **d**, **e** XPS spectra of Pb 4*f* and Br 3*d* peaks for PeQWs coated with and without TAPC and TBTB.

Blue, green and red PeLEDs based on PeQW arrays are fabricated with a device structure of aluminum (Al)/aluminum oxide (Al_2_O_3_)/PeQWs/TAPC/1,4,5,8,9,11-Hexaazatriphenylenehexacarbonitrile (HAT-CN)/ITO schematically shown in Fig. 3a. The thickness of Al_2_O_3_, PeQWs, TAPC, HAT-CN and ITO can be measured to be ~5, 90, 20, 20 and 80 nm, respectively from the cross-sectional SEM image in Fig. 3b. The flat-band energy diagram of PeLEDs is illustrated in Fig. 3c. The valance band maximum (VBM) of perovskite QWs is calculated from the ultraviolet photoelectron spectroscopy (UPS) spectra in Supplementary Fig. 13. In this structure, Al acts as a cathode and the ultrathin Al_2_O_3_ layer (~5 nm), whose existence can be verified by the HRTEM images in Supplementary Fig. 14, works as an insulating layer to block holes during the device operation^28^. The Al/Al_2_O_3_/PeQWs forms a metal-insulator-semiconductor (MIS) structure and its working mechanism has been clearly explained in other report^28,41^. The TAPC layer is used to passivate the top surface of PeQWs and transport the injected holes^42^, while the TBTB is doped to heal halide vacancy. The PL decay curves before and after the TBTB doped TAPC deposition are shown in Supplementary Fig. 15. As demonstrated in Fig. 3d, e, the peaks of Pb 4*f* and Br 3*d* shift slightly to lower binding energy after the deposition of TAPC on PeQWs, which can be ascribed to the change in the electron cloud density due to the Pb–N interactions and the change in the bond vibration, suggesting that TAPC has electrostatically passivated the PeQWs top surface^21,43,44^ and results in further suppression of the trap-mediated nonradiative recombination. At the same time, TBTB is introduced into TAPC to act as a halide vacancy healing agent. As mentioned in the previous report^7^, the TBTB molecule will provide bromide-rich environment to the perovskite surface and the bromine from the molecule can be readily deposited into a surface bromide vacancy. The introduction of TBTB will also lead to a shift in XPS spectrum. It is reported that the halide segregation is caused by the hopping of halide ions from one halide vacancy site to the nearby ones in the crystal lattice^45^. Since the halide vacancies on the top surface of PeQWs have been pronouncedly reduced by TAPC and TBTB, the halide segregation can be dramatically suppressed. A layer of 20 nm HAT-CN is thermally deposited on top of TAPC, serving as hole injection layer and buffer layer to reduce the surface damage caused by the subsequent ITO sputtering^46^.

**Fig. 4:**
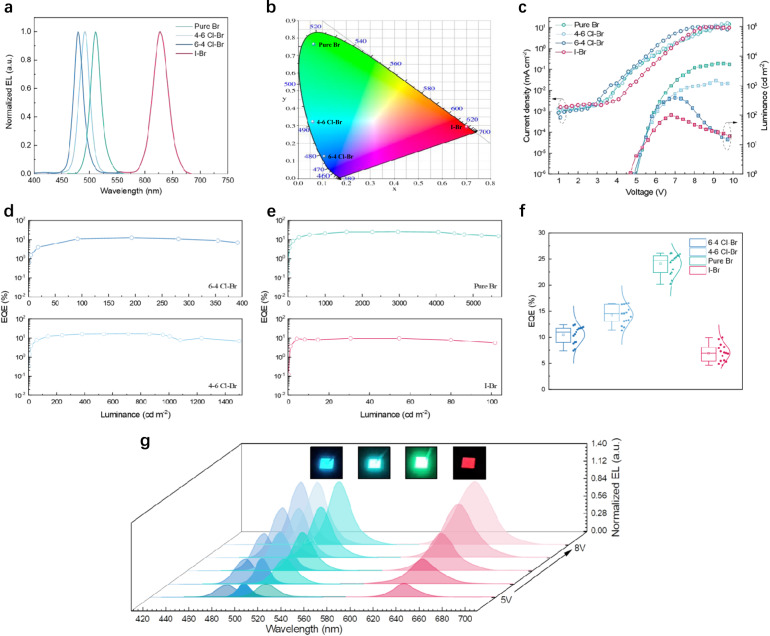
PeQWs-based LED device performance characteristics. **a** Normalized EL spectra of blue, sky-blue, green and pure-red PeQWs-based LEDs. **b** The corresponding CIE coordinates. **c**, **d**, Representative operational characteristics for our PeQWs-based LEDs. **c** Current density (*J*)–voltage (*V*) and luminance (*L*)–voltage (*V*) curves. **d**, **e** External quantum efficiency (EQE)–luminance (*L*) characteristics. **f** Distribution of EQEs of four PeQWs-based LEDs. **g** Electroluminescence spectra at different electric biases for blue, sky-blue, green and pure-red PeQWs-based LEDs. The insets show electroluminescence images of for blue, sky-blue, green and pure-red LEDs.

The EL spectrum and the corresponding Commission Internationale de l’éclairage (CIE) chromaticity coordinate diagram of blue, green and red PeQWs-based PeLEDs are shown in Fig. 4a, b, separately. The PL and EL peaks positions are congruent with each other, regardless of the slight broadening of the EL spectra. As can be seen from the CIE diagram, the emission color of four PeLEDs are positioned at the blue, sky-blue, green and pure-red regions. Figure 4c–e illustrates the current density (*J*)-luminance (*L*)-voltage (*V*) and external quantum efficiency (EQE)-luminance (*L*) characteristics of the corresponding PeLEDs. From the *J*–*L*–*V* and EQE–*L* curves, the best performing blue, sky-blue, green and pure-red PeLEDs have the peak EQE values of 12.41%, 16.49%, 26.09% and 9.97%, respectively, with a maximum luminance of 670 cd m^−2^, 1788 cd m^−2^, 12147 cd m^−2^ and 101 cd m^−2^ (Supplementary Fig. 16). Note that our device structure is top-emission structure which is rarely reported in the PeLEDs research community. However, top-emission device structure is highly preferred for display applications since it is best suited to active-matrix design. In fact, here we report the highest efficiency top-emission blue PeLED, to our best knowledge. Meanwhile, the 16.49% EQE for sky-blue color is the record for all-inorganic sky-blue PeLEDs. The 26.09% EQE for green LED is the record for all-inorganic PeLEDs and also the record for all top-emission PeLEDs so far. More impressively, the EQE histograms for four types of PeLEDs presented in Fig. 4f and Supplementary Fig. 17 show average EQEs of 10.47%, 14.24%, 24.13% and 6.97% with a small standard deviation of around 2%. Such high EQE values can be attributed to the free of current leakage, high crystal quality, quantum confinement effect induced high radiative recombination and inherent high light OCE. The finite-difference-time-domain (FDTD) simulations are performed to validate the fact that PeQWs-based LEDs have significantly improved OCE compared with planar PeLEDs. Generally, the OCE of planar PeLEDs without applying any light extraction strategy ranges from 10% to 20%^47,48^. In comparison, the OCE of PeLEDs based on PeQW arrays can be as high as 82.06% which is 4 times higher than planar ones (Supplementary Fig. 18 and Supplementary Table 1). The EL spectra under different voltages and photographs of working PeLEDs are shown in Fig. 4g. As the applied voltage increases to 7–10 V, the EL intensity gradually reaches the highest value and the EL peak position remains almost unchanged, manifesting that the light emission from our PeLEDs is spectrally stable. When high voltage (>10 V) was applied, the EL peak position for all devices still experiences no obvious shifting (Supplementary Fig. 19). The EL full width at half maximum (FWHM) broadening is the concerted effect caused by a field-induced quantum confinement Stark effect (QCSE), larger exciton polarization and Joule heating under high electrical bias which is mentioned in other report^49^. The excellent spectral stability is achieved from the thwarted halide segregation due to the introduction of rubidium ions, the relatively high compositional homogeneity of PeQWs as well as passivation from TAPC, TBTB and Al_2_O_3_. The lifetime tests carried out in ambient condition without encapsulation (relative humidity of 50–70%) are summarized in Supplementary Fig. 20. Although high spectral stability has been achieved, the issue relating to the long-term stability in terms of luminance and efficiency still requires more efforts in the future.

**Fig. 5:**
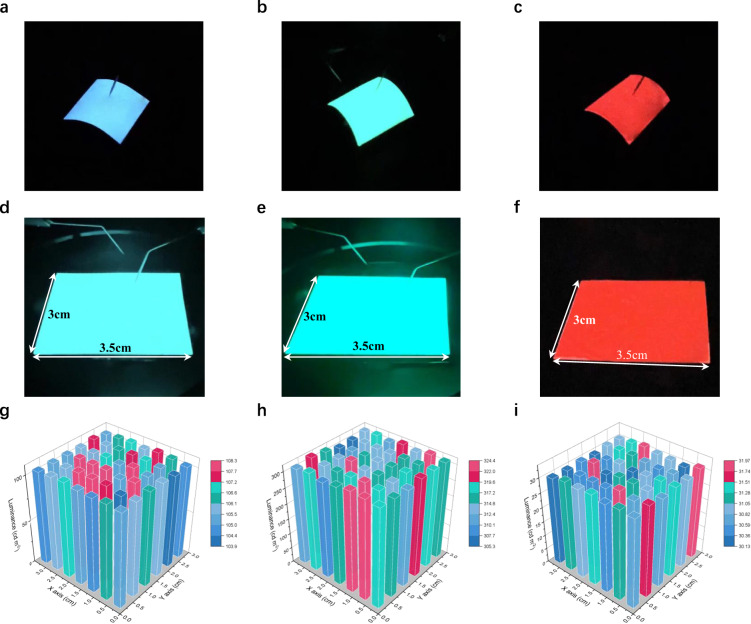
Flexible and large-area PeQWs-based PeLED device. **a**–**c** The flexible PeQWs-based LEDs with an electroluminescent area of 1.5 × 1.5 cm^2^. **d**–**f** Photograph of large-area PeQWs-based LEDs with a device area of 3 × 3.5 cm^2^. **g**–**i** Luminance distribution of the PeQWs-based LEDs (3 × 3.5 cm^2^) under working conditions. Luminance is presented using a 6 × 7-pixel 3D column chart.

Finally, to validate the versatility of the unique and universal strategy proposed here, flexible and large-area PeQWs-based LED devices with sky-blue, green and pure-red emissions are fabricated, further demonstrating the promise of portable applications which require the devices to be in flexible form. Figure 5a–c present the flexible sky-blue, green and red devices (1.5 × 1.5 cm^2^) with uniform EL measured under bending. The rigid devices with the same area are shown in Supplementary Fig. 21a–c. To further demonstrate the upscaling capability, larger-area devices with functional area up to 2 × 2.5 cm^2^ and 3 × 3.5 cm^2^ are fabricated (Supplementary Fig. 22a–c and Fig. 5d–f). The luminance from large-area devices at different positions are measured and summarized by the 3D column chart. The large-area LED devices exhibit uniform EL distribution with small deviation (Supplementary Figs. 21d–f, 22d–f and Fig. 5g–i).

## Discussion

In conclusion, we have demonstrated a generic organic ligand-free and antisolvent-free solution method to grow crystalline all-inorganic PeQW arrays in nanoporous PAM templates with excellent uniformity. The obtained PeQW arrays simultaneously possess high crystal quality, high PLQY and high light OCE. By introducing a dual-functional small molecule TAPC on top of PeQWs, serving as passivation and hole transporting layer, and a halide vacancy healing agent TBTB, the trap-mediated nonradiative recombination has been significantly suppressed. The PeQW arrays are then integrated in LED devices which deliver record high champion EQEs of 12.41%, 16.49%, 26.09% and 9.97% for top-emission blue, sky-blue, green and pure-red all-inorganic PeLEDs, respectively. The EL spectra from PeQWs-based LEDs show no obvious change under high electrical bias, signifying halide segregation has been effectively suppressed. We believe that high luminance and long-term stability can be achieved in the future by dopant engineering to suppress the ion migration and device thermal management. Taking advantage of ultrahigh density and excellent uniformity of the PeQW arrays, we also achieved uniform emission from flexible and large-area devices. We anticipate our strategy provides a universal way to realize high-performance PeLEDs for future wide-color-gamut displays and solid-state lighting.

## Methods

### Materials

Cesium bromide (CsBr, 99.999%), rubidium bromide (RbBr, 99.6%), lead bromide (PbBr_2_, 99.999%), lead chloride (PbCl_2_, 99.999%), lead iodide (PbI_2_, 99.999%), molybdenum trioxide (MoO_3_, ≥99.5%), 1,3,5-tris(bromomethyl)−2,4,6-triethylbenzene (TBTB, 97%), chlorobenzene (C_6_H_5_Cl, 99.8%) and dimethyl sulfoxide (DMSO, ≥99.9%) were purchased from Sigma Aldrich. 1,1-Bis[(di-4-tolylamino)phenyl]cyclohexane (TAPC, >99.5%) and 1,4,5,8,9,11-Hexaazatriphenylenehexacarbonitrile (HAT-CN, >99%) were purchased from Ossila. All materials were used as received without further purification.

### Preparation of the perovskite solution

All perovskite precursors were prepared by dissolving the chemicals into DMSO. For green emitting perovskite solution, RbBr, CsBr and PbBr_2_ were mixed and dissolved in DMSO with a molar ratio of 0.1:1.4:1. For blue emitting perovskite solutions, RbBr, CsBr, PbBr_2_ and PbCl_2_ were mixed and dissolved in DMSO with a molar ratio of 0.1:1.4: *x*:*y* (where *x* + *y* = 1). The chloride concentration is defined as *n*_Cl_ = *y*/(*x* + *y*). For red emitting perovskite solution, RbBr, CsBr, PbBr_2_ and PbI_2_ were mixed and dissolved in DMSO with a molar ratio of 0.1:1.2: *x*:*z* (where *x* + *z* = 1). The iodide concentration is defined as *n*_I_ = *z*/(*x* + *z*). The concentration for all perovskite solutions determined by Pb^2+^ is 0.1 M. The precursor solutions were stirred at 60 °C for 12 h and filtered by 0.45-μm poly(tetrafluroethylene) filters before use.

### PAM templates fabrication

The PAM templates were fabricated by anodic anodization of high purity aluminum foil under low voltage which can be found in our previous work^12^. In brief, flexible and rigid aluminum foils with different areas were electro-polished in an acidic solution (25 vol% perchloric acid and 75 vol% absolute ethanol) at 15 V for 4 min at room temperature. Subsequently, the polished aluminum foils were anodized in a solution of 5 vol% H_2_SO_4_ at 5 V for 8 min to form 85-nm-thick porous aluminum oxide with 6.4 nm pore diameter. Finally, the PAM templates were rinsed with deionized water and dried by compressed air. For flexible PAM templates, thin (100 μm) and flexible PAM templates were attached on the PET substrate.

### Perovskite thin film deposition

ITO substrates were consecutively sonicated with detergent, deionized water, acetone and isopropyl alcohol for 10 min, respectively. After being dried by compressed air, the clean substrates were treated by oxygen plasma for 10 min. The substrates were then transferred into a nitrogen-filled glovebox. For perovskite thin film, the perovskite precursor solutions were spin-coated at 3000 rpm for 30 s, followed by baking at 80 °C for 10 min.

### Small-area PeLEDs fabrication

After the fabrication of PAM templates, the clean 2 × 2.5 cm^2^ PAM templates were transferred into a nitrogen-filled glovebox for perovskite deposition. The perovskite precursor solutions were deposited by spin-coating at 6000 rpm for 50 s, followed by annealing at 80 °C for 10 min. This process allowed the formation of high-density PeQW arrays without bulk perovskite layer on the PAM template surface. The TAPC solution doped with TBTB (in chlorobenzene) was spin-coated on PAM templates filled with perovskite at 3000 rpm for 40 s. Finally, 20 nm HAT-CN was thermally evaporated (1 Å s^−1^) as buffer layer and 80 nm ITO was magnetically sputtered (1 Å s^−1^) at 50 W by RF sputtering to complete the whole fabrication. The device area was 0.04 cm^2^ defined by a shadow mask which was used during the ITO sputtering.

### Flexible and large-area PeLEDs fabrication

The clean flexible and large-area PAM templates were transferred into a nitrogen-filled glovebox for perovskite deposition. After the deposition of perovskite, the substrates were then transferred into a vacuum chamber. 25 nm TAPC and 20 nm HAT-CN were thermally evaporated on top of the substrates, respectively. Finally, ITO was magnetically sputtered with a thickness of 80 nm. The device areas were 1.5 × 1.5 cm^2^, 2 × 2.5 cm^2^, 3 × 3.5 cm^2^, respectively.

### Characterization

Top-view SEM images were taken by a Dual Beam FIB/SEM system (FEI Helios G4 UX). TEM images were obtained from a transmission electron microscope (TEM) JEOL (2010F). XRD patterns were recorded on a Bruker D8 X-ray diffractometer. The PL spectrum, PLQY and TRPL were measured using an Edinburgh Instruments FLS920P. The TA absorption spectrum was collected from a Helios (Ultrafast Systems LLC) spectrometers equipping a sapphire laser source (Coherent Legend, 800 nm, 150 fs, 5 mJ per pulse, and 1 kHz repetition rate). Seventy-five percent of the 800 nm output pulse was frequency-doubled by a BaB_2_O_4_ (BBO) crystal, generating 400 nm pump light. At the same time, 25% of the output pulse was concentrated into a sapphire window to produce white light continuum (420–780 nm) probe light. The 400 nm pump beam with a beam waist of about ∼360 μm was focused on the sample and the power intensity was fixed at 40 μJ cm^−2^. XPS and UPS spectrum were characterized from a Kratos Axis Ultra DLD multi-technique surface analysis system.

### PeLEDs measurement

The PeLEDs were driven by a Keithley 2450 source-meter as a voltage source in ambient air without encapsulation. The luminance, CE and EQE were collected with an Ocean Optics Flame spectrometer and an integrating sphere. Calibration of the spectrometer was done as reported in our previous work^50^. The luminance was cross-checked using a luminance meter (Konica Minolta, CS-200).

## Supplementary information

Supplementary Information

Peer Review File

## Data Availability

The data that support the findings of this study are provided in the main text and the Supplementary Information. More data are available from the corresponding author upon request.
