# Supplementary material for: High-efficiency, flexible and large-area red/green/blue all-inorganic metal halide perovskite quantum wires-based light-emitting diodes
Source: Nat Commun. 2023 Aug 1;14:4611. doi: 10.1038/s41467-023-40150-y (PMC10393990; doi:10.1038/s41467-023-40150-y)
Supplement: Supplementary file 1 — Supplementary Information [file 41467_2023_40150_MOESM1_ESM.pdf]

Supplementary Information for:

High-Efficiency, Flexible and Large-area Red/Green/Blue All-Inorganic Metal Halide Perovskite Quantum Wires-Based Light-Emitting Diodes

Yang Bryan Cao[1,2,3], Daquan Zhang[1,2,3], Qianpeng Zhang[1,2,3], Xiao Qiu[1,2,3], Yu Zhou[1,2,3], Swapnadeep Poddar[1,2,3], Yu Fu[1,2,3], Yudong Zhu[1,4], Jin-Feng Liao[5], Lei Shu[1,2,3], Beitao Ren[1,2,3], Yucheng Ding[1,2,3], Bing Han[4], Zhubing He[4], Dai-Bin Kuang[5], Kefan Wang[6], Haibo Zeng[7], Zhiyong Fan[1,2,3*]*

[1]Department of Electronic & Computer Engineering, The Hong Kong University of Science and Technology, Clear Water Bay, Kowloon, Hong Kong SAR, China.

[2]State Key Laboratory of Advanced Display and Optoelectronics Technologies HKUST, Clear Water Bay, Kowloon, Hong Kong SAR, China.

[3]Guangdong-Hong Kong-Macau Joint Laboratory for Intelligent Micro-Nano Optoelectronic Technology, HKUST, Clear Water Bay, Kowloon, Hong Kong SAR, China.

[4]Department of Materials Science and Engineering, Southern University of Science and Technology, No. 1088, Xueyuan Rd., Shenzhen, 518055, Guangdong, P. R. China.

[5]MOE Key Laboratory of Bioinorganic and Synthetic Chemistry, Lehn Institute of Functional Materials, School of Chemistry, Sun Yat-sen University, Guangzhou 510275, P. R. China.

[6] Henan Provinces Key Laboratory of Photovoltaic Materials, Henan University, Kaifeng 475004, Henan, China.

[7]MIIT Key Laboratory of Advanced Display Materials and Devices, Institute of Optoelectronics & Nanomaterials, School of Materials Science and Engineering, Nanjing University of Science and Technology, Nanjing 210094, China.

*Corresponding author: eezfan@ust.hk (Z.F.), zeng.haibo@njust.edu.cn (H.Z.).

This file include:

Supplementary Figure 1-22

Supplementary Table 1-3

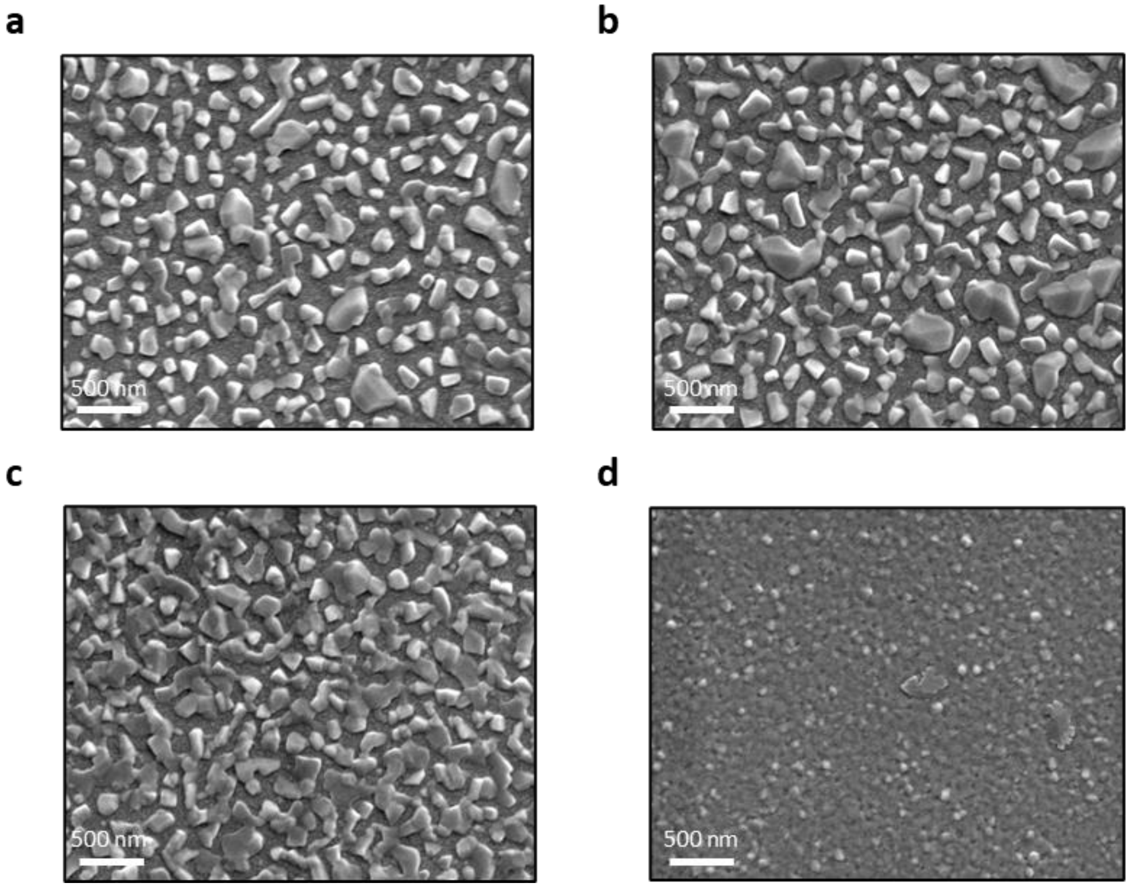

Supplementary Figure 1. Top-view scanning electron microscopy (SEM) images of perovskite films deposited. a, 6-4 Cl-Br perovskite. **b**, 4-6 Cl-Br perovskite. **c**, $CsPbBr_3$. **d**, I-Br perovskite. All the films were deposited on ITO glass.

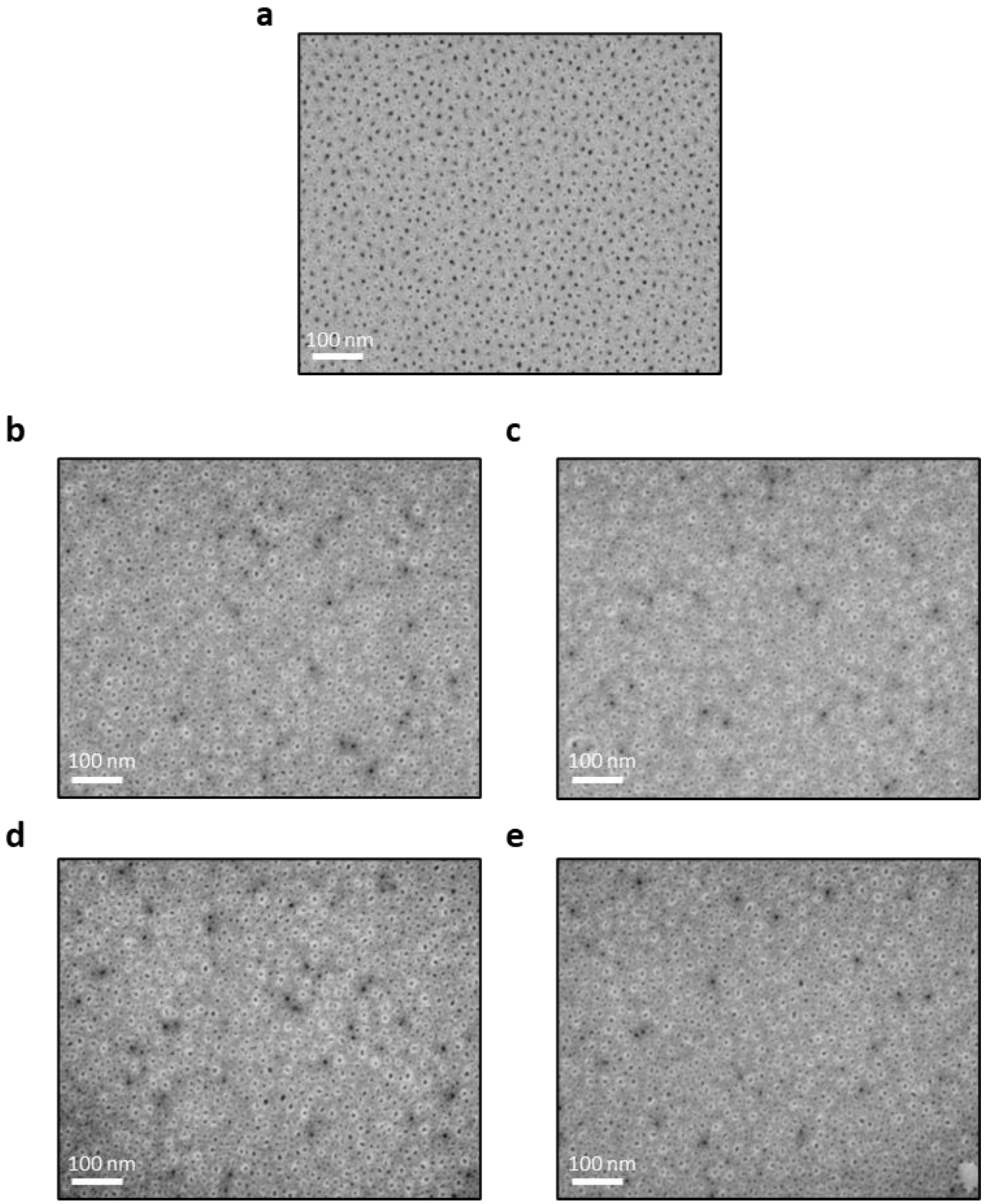

Supplementary Figure 2. Top-view SEM images of perovskite QWs. a, blank PAM subtrate **b**, 6-4 Cl-Br PeQWs. **c**, 4-6 Cl-Br PeQWs. **d**, $CsPbBr_3$ QWs. **e**, I-Br PeQWs.

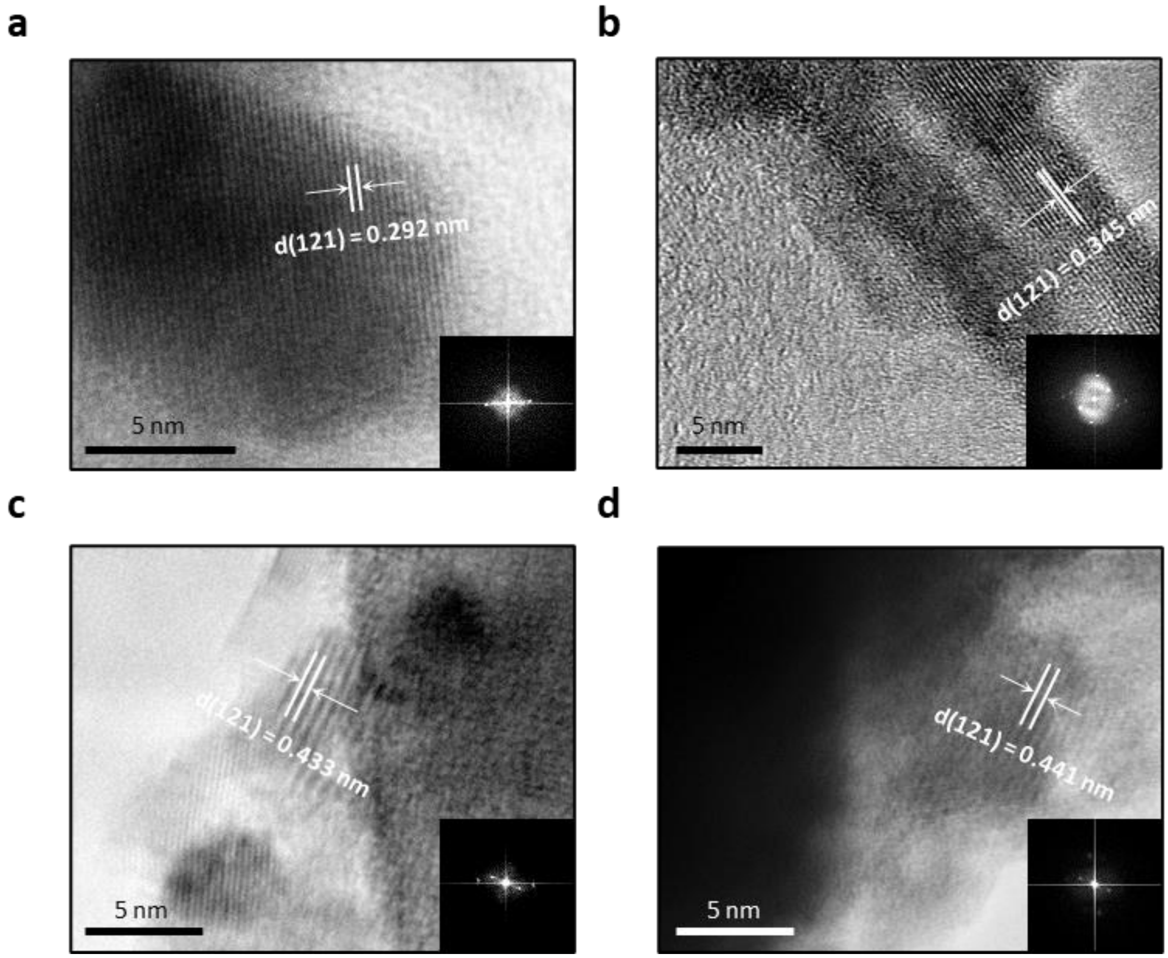

Supplementary Figure 3. High-resolution transmission electron microscopy (HRTEM) image of perovskite QWs. a, 6-4 Cl-Br perovskite. **b**, 4-6 Cl-Br perovskite. **c**, $CsPbBr_3$. **d**, I-Br perovskite.

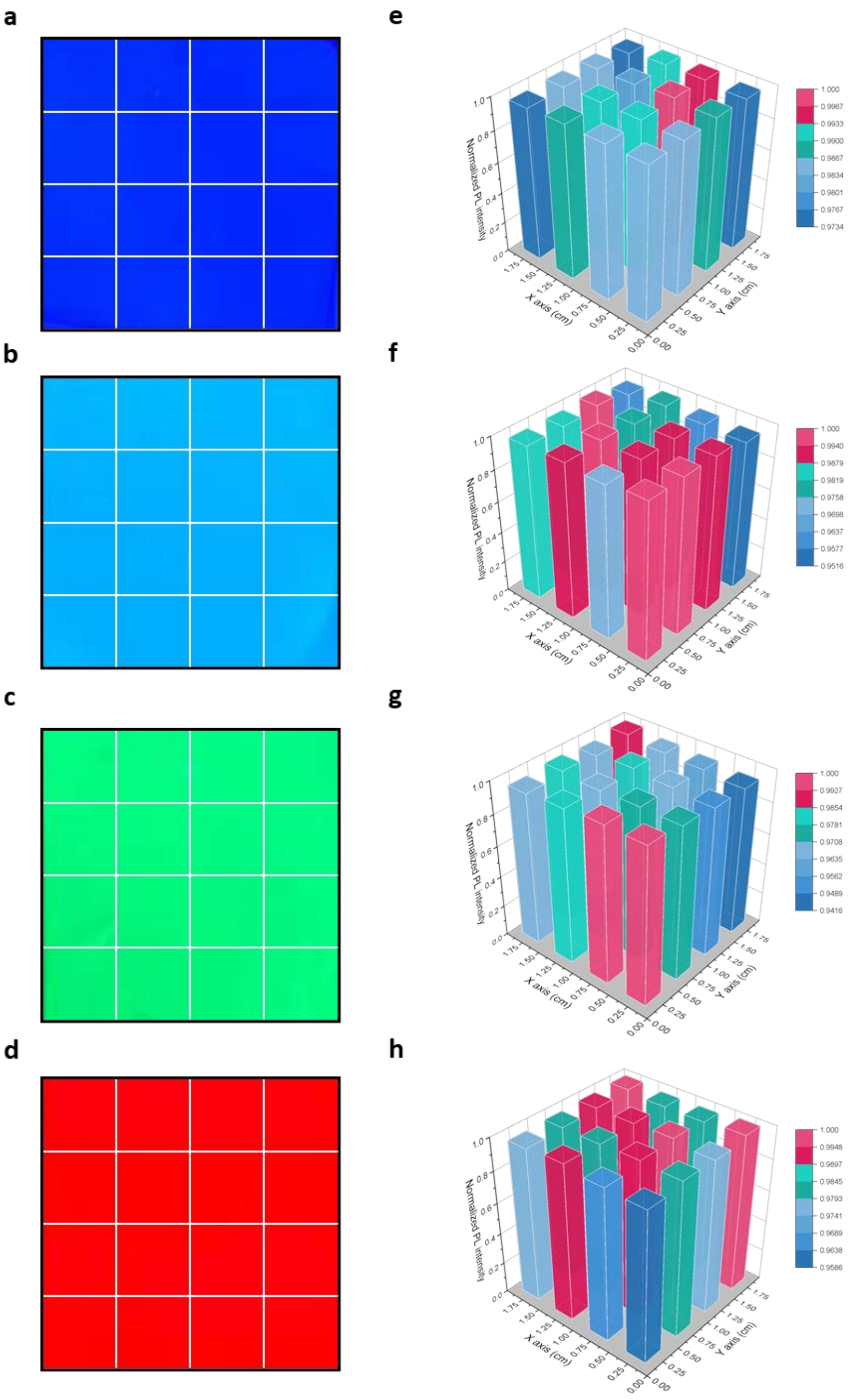

Supplementary Figure 4. Photoluminescent intensity distribution of PeQWs arrays. a-d, Photograph of blue, sly-blue, green and pure-red light emitting PeQWs with an area of 2×2 cm^2 arrays. **e-h,** Normalized photoluminescent intensity of the PeQWs arrays with an area of 2×2 cm^2. The normalized photoluminescent intensity is presented by a 4×4-pixel 3D column chart.

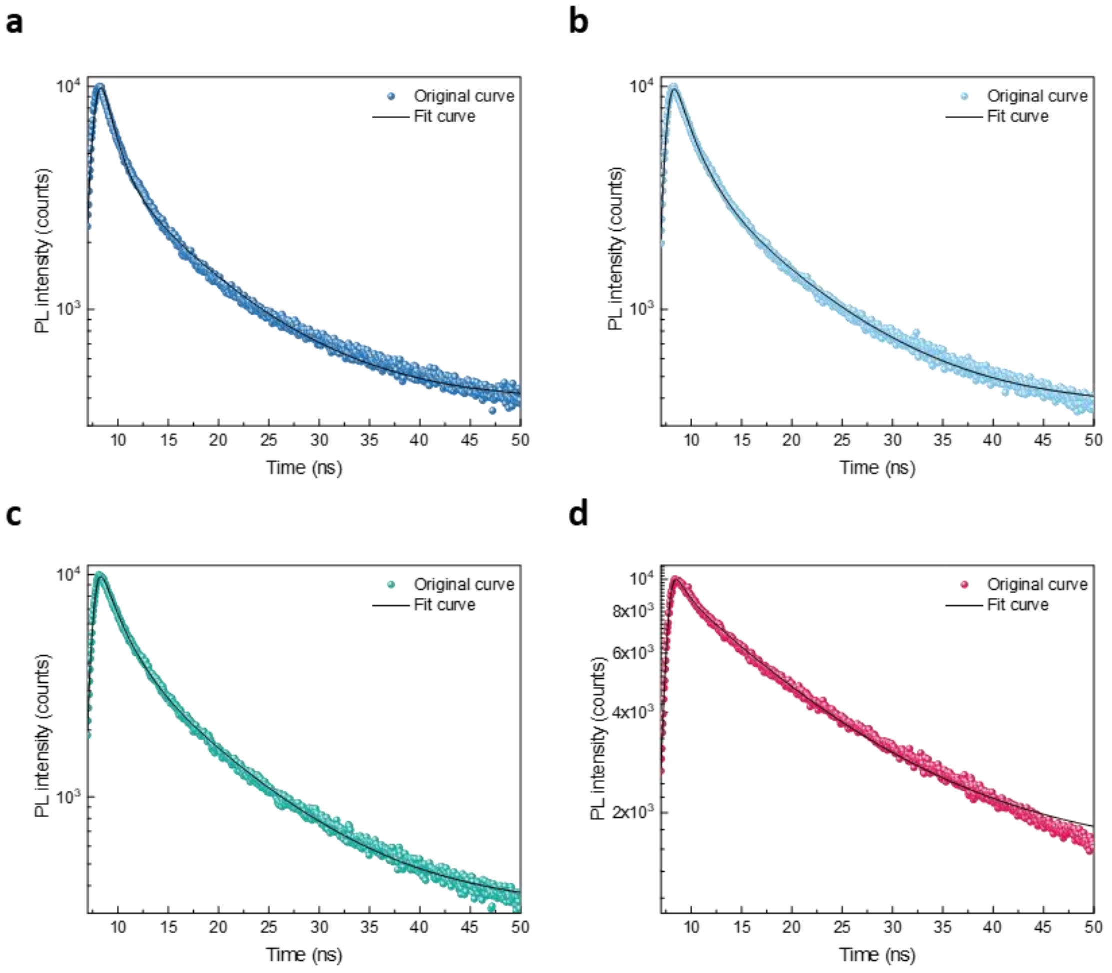

Supplementary Figure 5. Photoluminescence lifetime characteristics. Unfitted and fitted time-resolved photoluminescence decay curves for **a**, 6-4 Cl-Br PeQWs, **b**, 4-6 Cl-Br PeQWs, **c**, $CsPbBr_3$ QWs and **d**, I-Br PeQWs with and without TAPC.

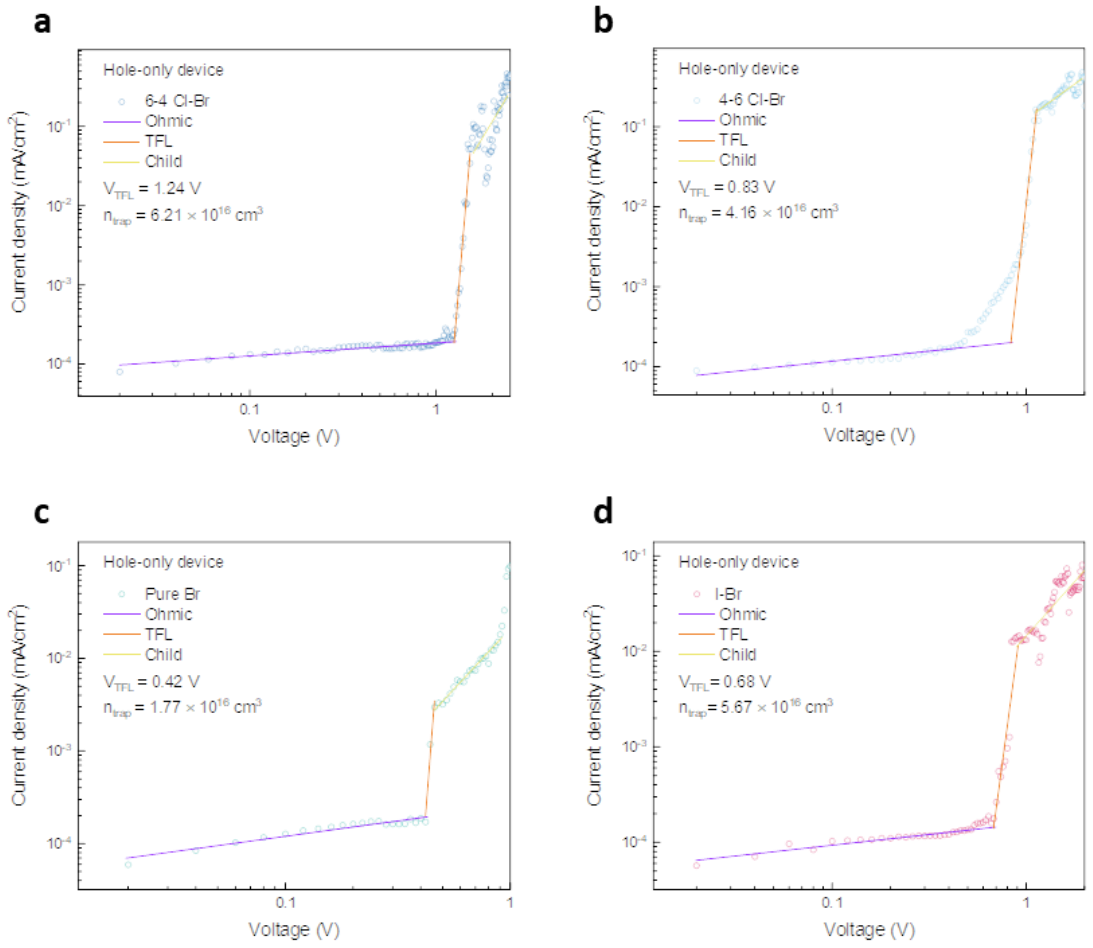

Supplementary Figure 6. The J−V characteristics of holy devices. The space-charge-limited current (SCLC) curves of a, 6-4 Cl-Br PeQWs. b, 4-6 Cl-Br PeQWs. c, $CsPbBr_3$ QWs. d, I-Br PeQWs.

To analyze the hole trap density of our PeQWs, Space charge-limited current (SCLC) measurement using a hole-only device structure is carried out. The total hole trap state density inside the PeQWs is calculated by the equation as below:

$$n_t = \frac{2\varepsilon\varepsilon_0 V_{TFL}}{eL^2}$$

where n_t is the trap state density, V_{TFL} is the trap-filled limit voltage, L is the thickness of the perovskites, e is the elementary charge, ε_0 and ε are the vacuum permittivity and relative permittivity, respectively.

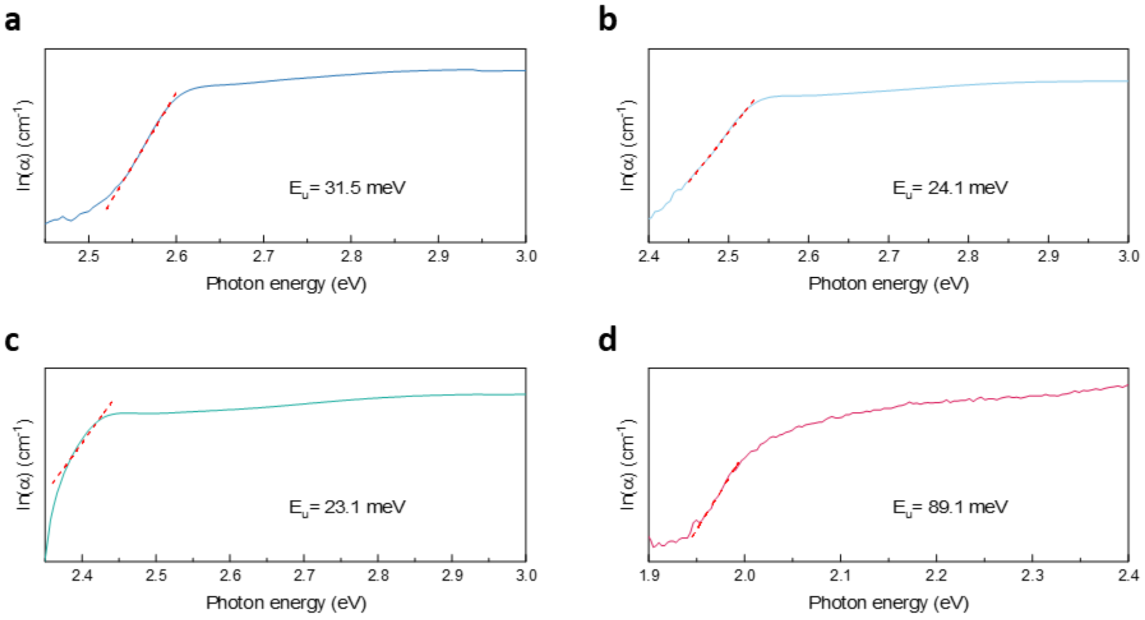

Supplementary Figure 7. The Urbach energy of perovskite QWs. Absorption coefficient spectra for **a,** 6-4 Cl-Br PeQWs. **b**, 4-6 Cl-Br PeQWs. **c**, CsPbBr3 QWs. **d**, I-Br PeQWs.

The absorption coefficient spectrum is extracted from the absorbance spectrum. In this way, the Urbach energy can be calculated as follow:

$$\alpha = \alpha_0 e^{\frac{h\nu}{E_u}}$$

where α is the absorption coefficient and $h\nu$ is the photon energy. The Urbach energy was found to be 23.1 meV, 24.1 meV, 31.5 meV and 89.1 meV for 6-4 Cl-Br, 4-6 Cl-Br, $CsPbBr_3$ and I-Br PeQWs, respectively. Urbach energy is a parameter used to quantify energetic disorder in the band edges of a semiconductor. The low Urbach energy indicates a low electronic disorder in the band edges for our PeQWs.

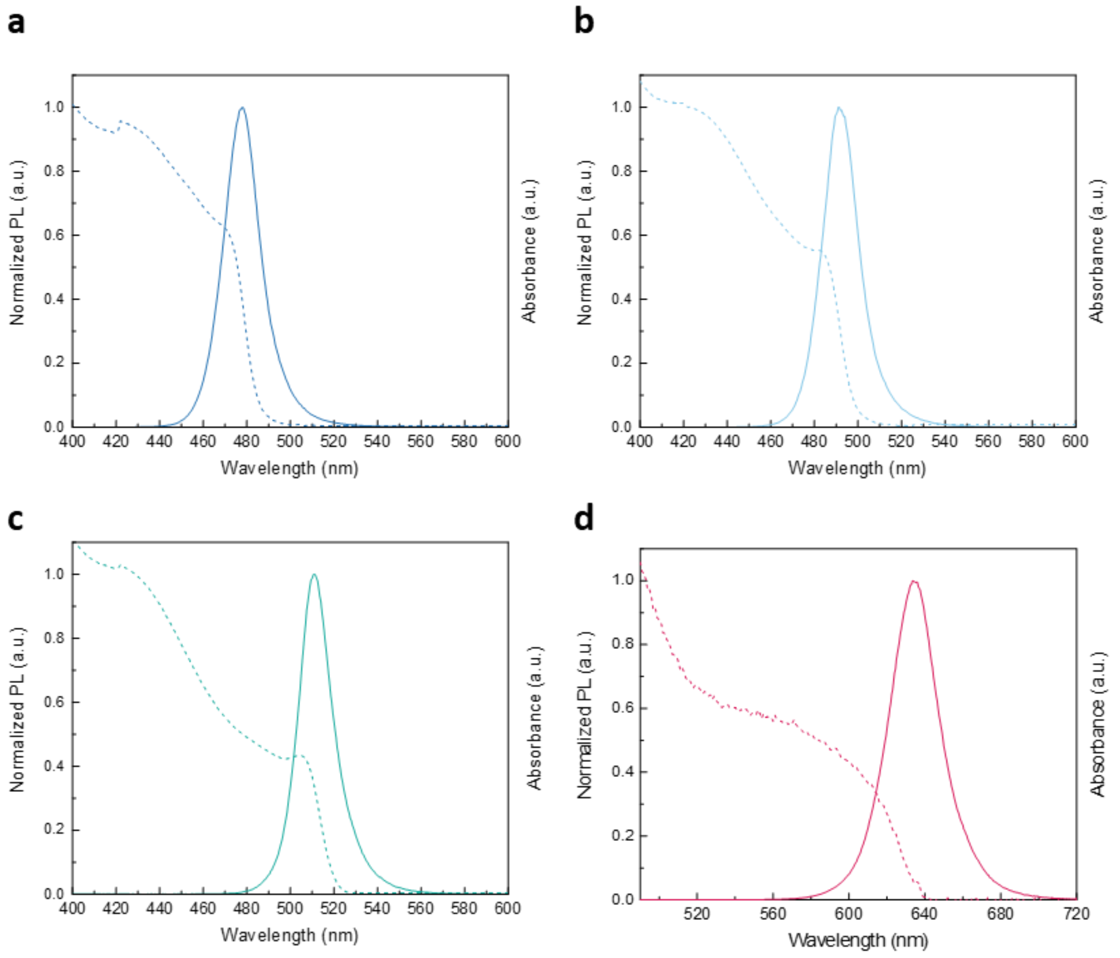

Supplementary Figure 8. Photoluminescence and absorbance of perovskite QWs. Photoluminescence and absorbance spectrum of **a**, 6-4 Cl-Br PeQWs. **b**, 4-6 Cl-Br PeQWs. **c**, $CsPbBr_3$ QWs. **d**, I-Br PeQWs.

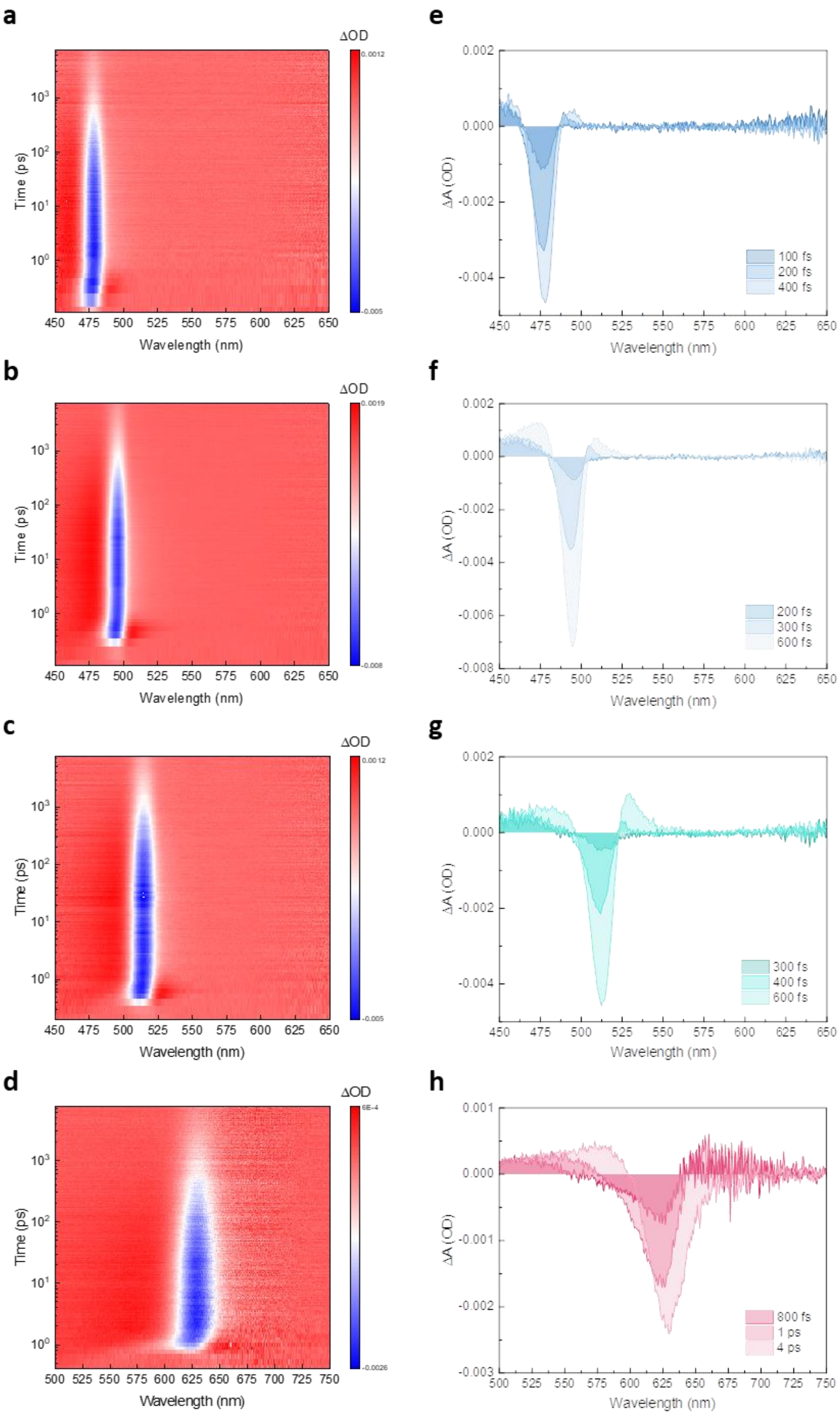

Supplementary Figure 9. Transient absorption (TA) spectra of perovskite QWs. Transient absorption of **a**,**e**, 6-4 Cl-Br PeQWs. **b**,**f**, 4-6 Cl-Br PeQWs. **c**,**g**, $CsPbBr_3$ QWs. **d**,**h**, I-Br PeQWs.

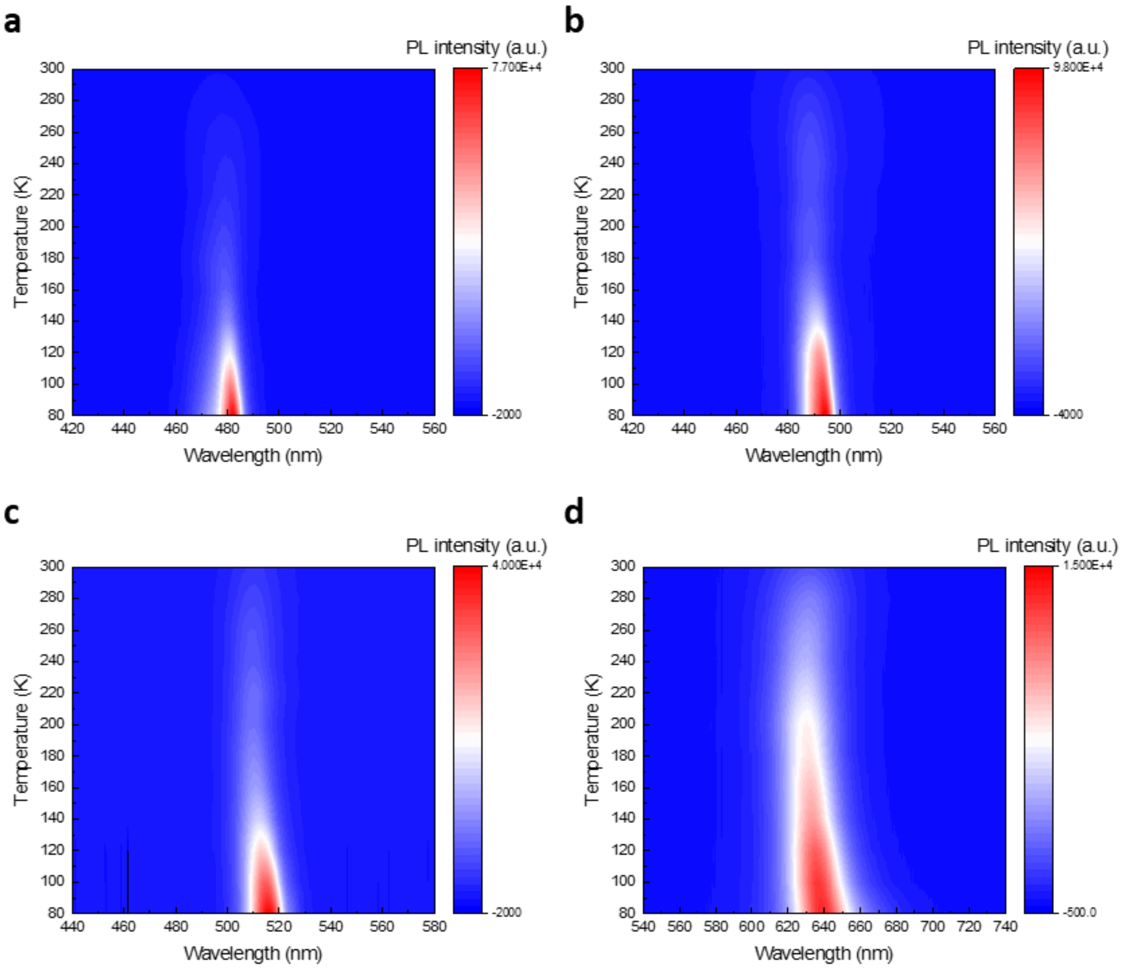

Supplementary Figure 10. Temperature-dependent PL spectra of perovskite QWs. Temperature-dependent PL spectra of **a**, 6-4 Cl-Br PeQWs. **b**, 4-6 Cl-Br PeQWs. **c**, $CsPbBr3$ QWs. **d**, I-Br PeQWs.

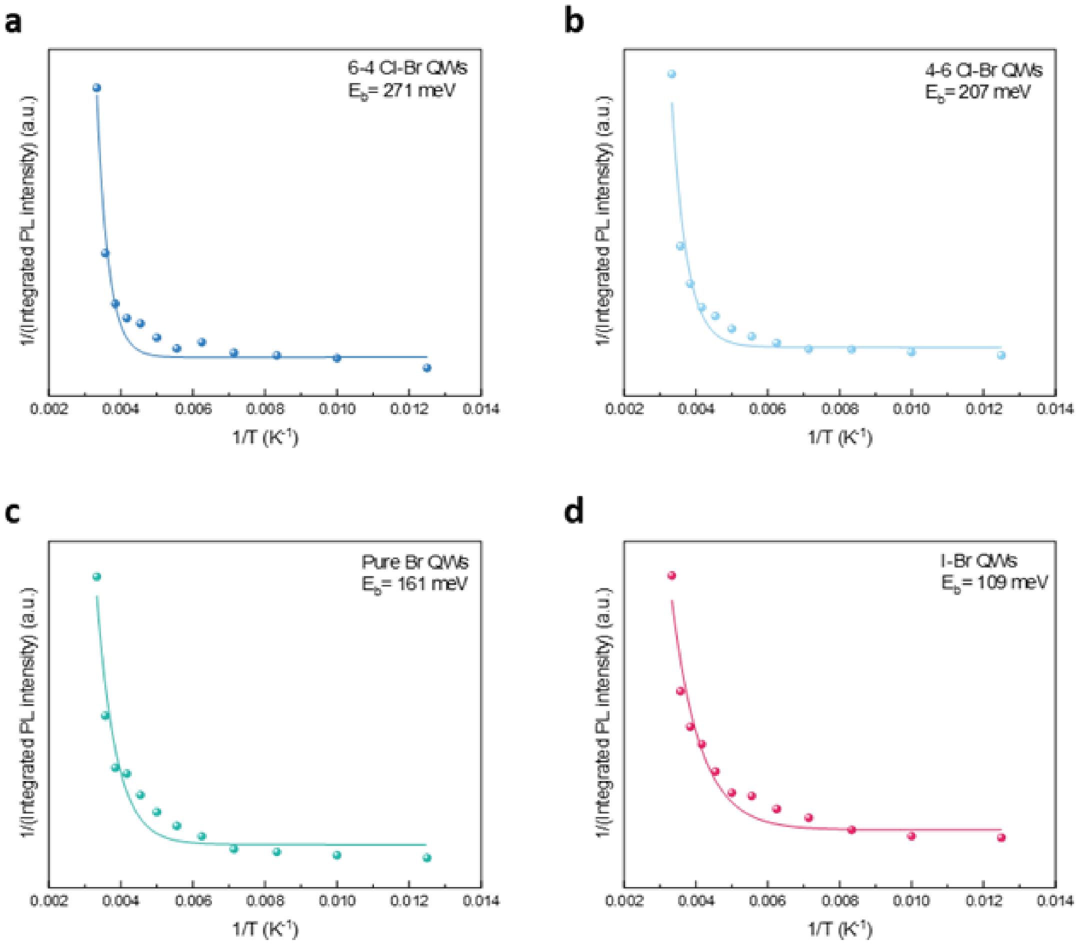

Supplementary Figure 11. The exciton binding energy of perovskite QWs. The calculated exciton binding energy of **a**, 6-4 Cl-Br PeQWs. **b**, 4-6 Cl-Br PeQWs. **c**, CsPbBr3 QWs. **d**, I-Br PeQWs.

The estimate of exciton binding energy by the temperature dependence of integrated PL signal is obtained by the equation as below:

$$I(T) = \frac{I_0}{1 + Ae^{-\frac{E_b}{k_B T}}}$$

where I_0 is the integrated PL intensity extrapolated at 0 K, A is a constant, E_b is the exciton binding energy, and k_b is the Boltzmann constant. Note that the exciton binding energy of perovskite might be over-estimated due to the possibility of structural transition at low temperature.

As a result, the exciton binding energy is approximately 271 meV, 207 meV, 161 meV and 106 meV for 6-4 Cl-Br, 4-6 Cl-Br, $CsPbBr_3$ and I-Br PeQWs, respectively.

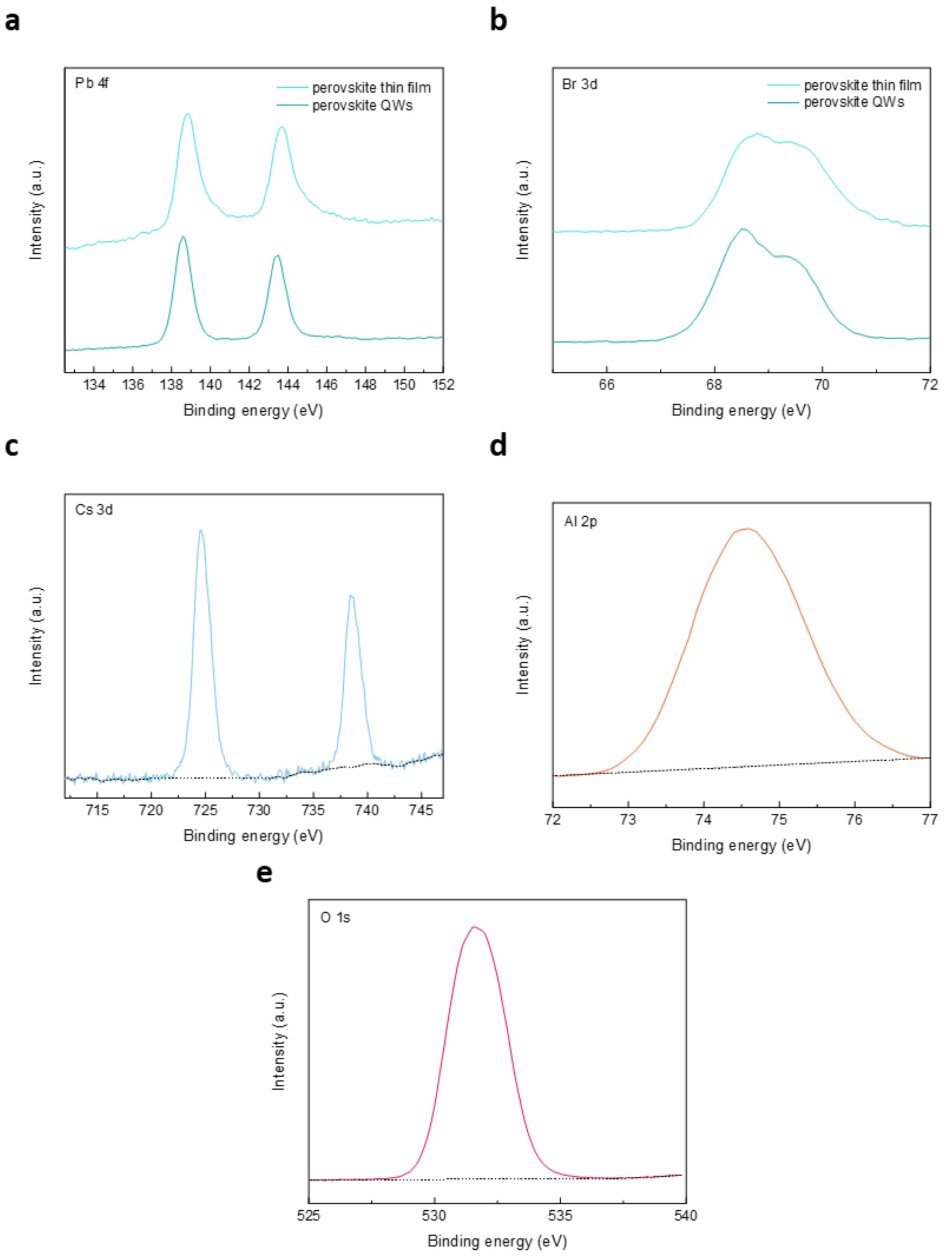

Supplementary Figure 12. X-ray photoelectron spectroscopy (XPS) spectra of perovskite thin films and QWs. a,**b**, Pb 4f and Br 3d spectra of perovskite thin films and QWs. XPS spectra for **c**, Cs 3d, **d**, Al 2p and **e**, O 1s.

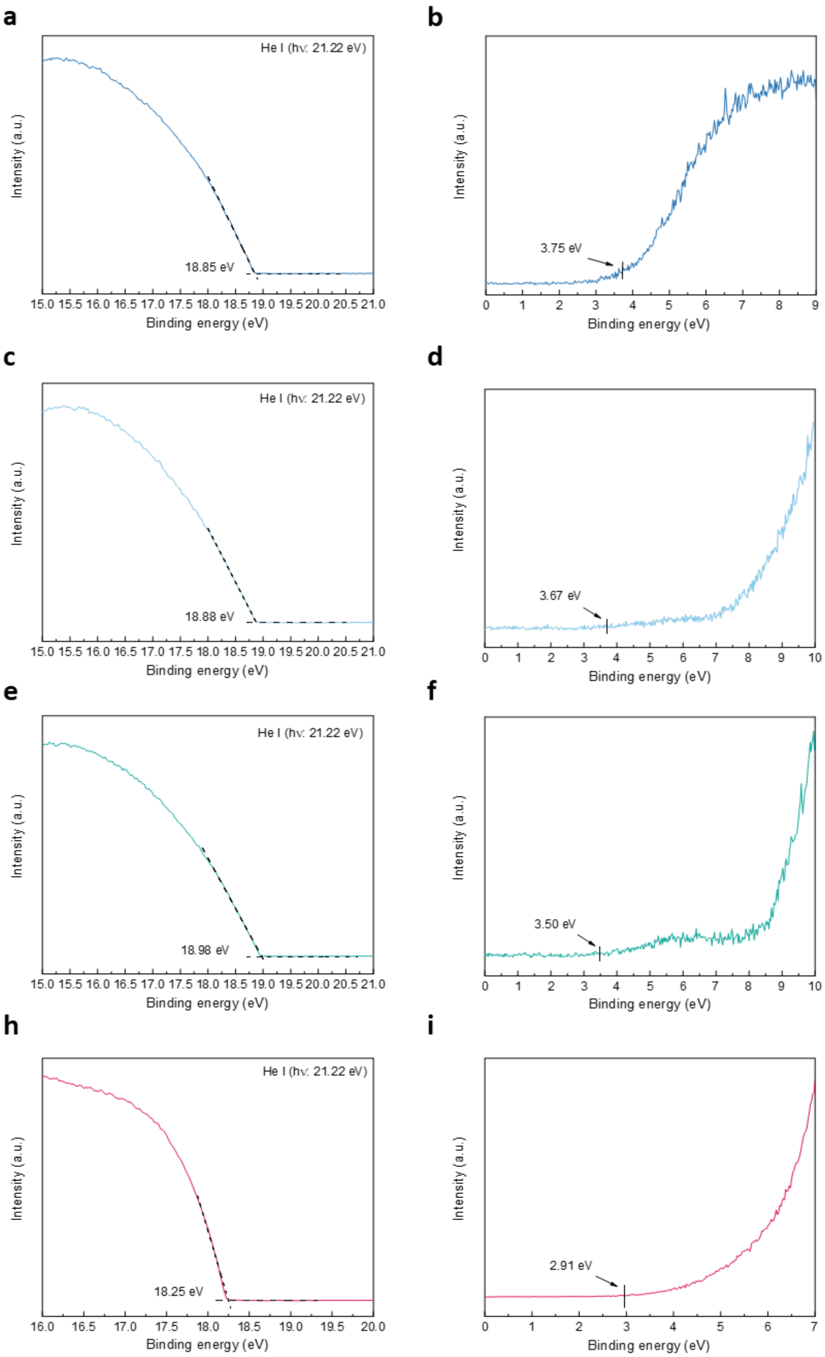

Supplementary Figure 13. Ultraviolet photoelectron spectroscopy (UPS) characteristics. Photoemission cutoff energy of **a**, 6-4 Cl-Br PeQWs, **c**, 4-6 Cl-Br PeQWs, **e**, $CsPbBr_3$ QWs, **h**, I-Br PeQWs and the valence-band region of **b**, $CsPbBr_3$ QWs, **d**, 4-6 Cl-Br PeQWs, **f**, 6-4 Cl-Br PeQWs, **i**, I-Br PeQWs from ultraviolet photoemission spectra.

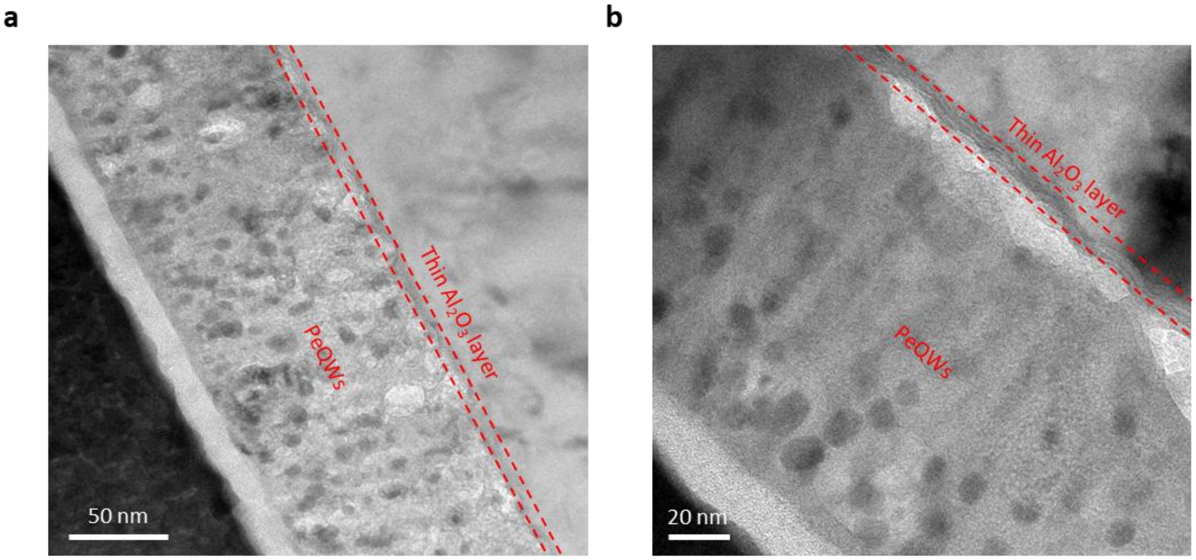

Supplementary Figure 14. Cross-sectional HRTEM image of perovskite QWs with ultra-thin Al_2O_3 layer. a and **b,** Cross-sectional HRTEM image showing perovskite QWs and ultra-thin Al_2O_3 layer**.** From the image, we can tell that the thickness of Al_2O_3 layer is around 5 nm.

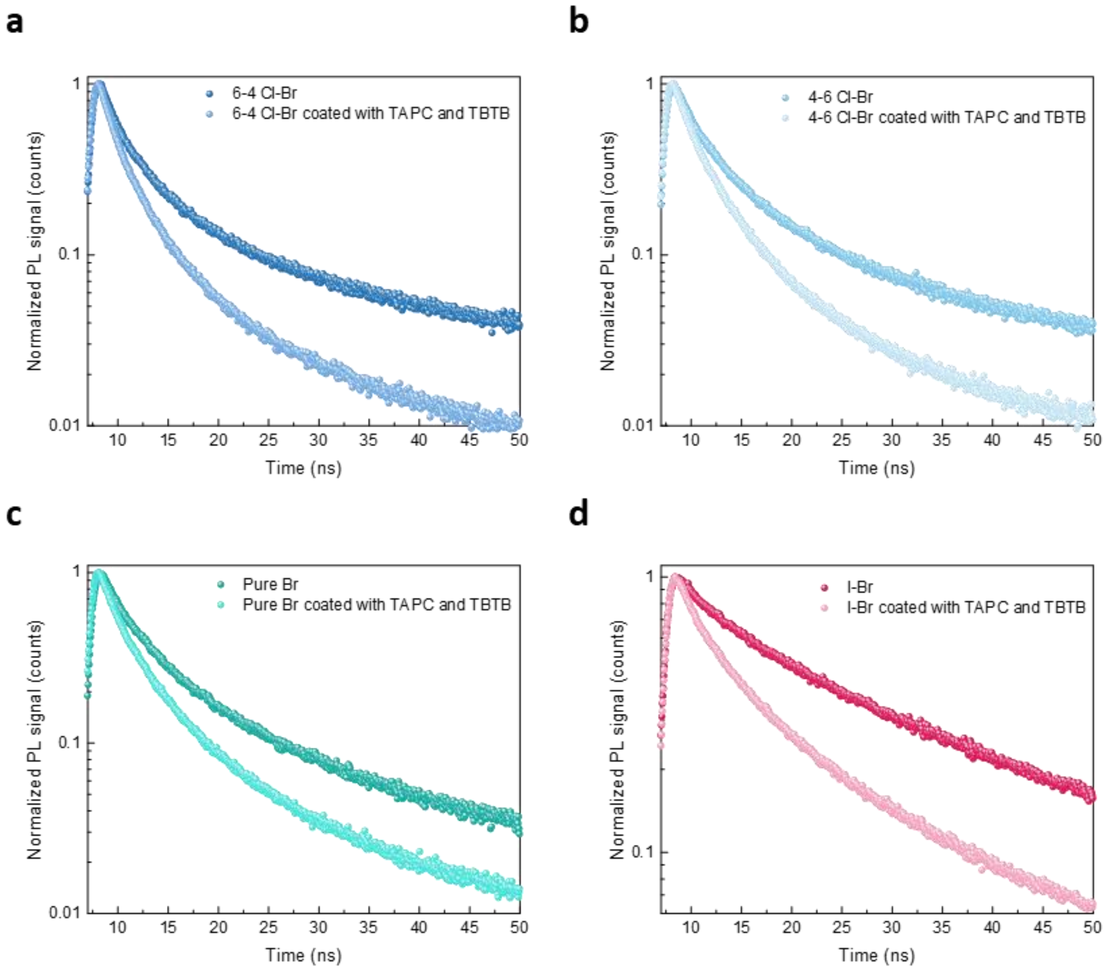

Supplementary Figure 15. Photoluminescence lifetime characteristics. Normalized time-resolved photoluminescence decay curves for **a**, 6-4 Cl-Br PeQWs, **b**, 4-6 Cl-Br PeQWs, **c**, $CsPbBr_3$ QWs and **d**, I-Br PeQWs with and without TAPC and TBTB.

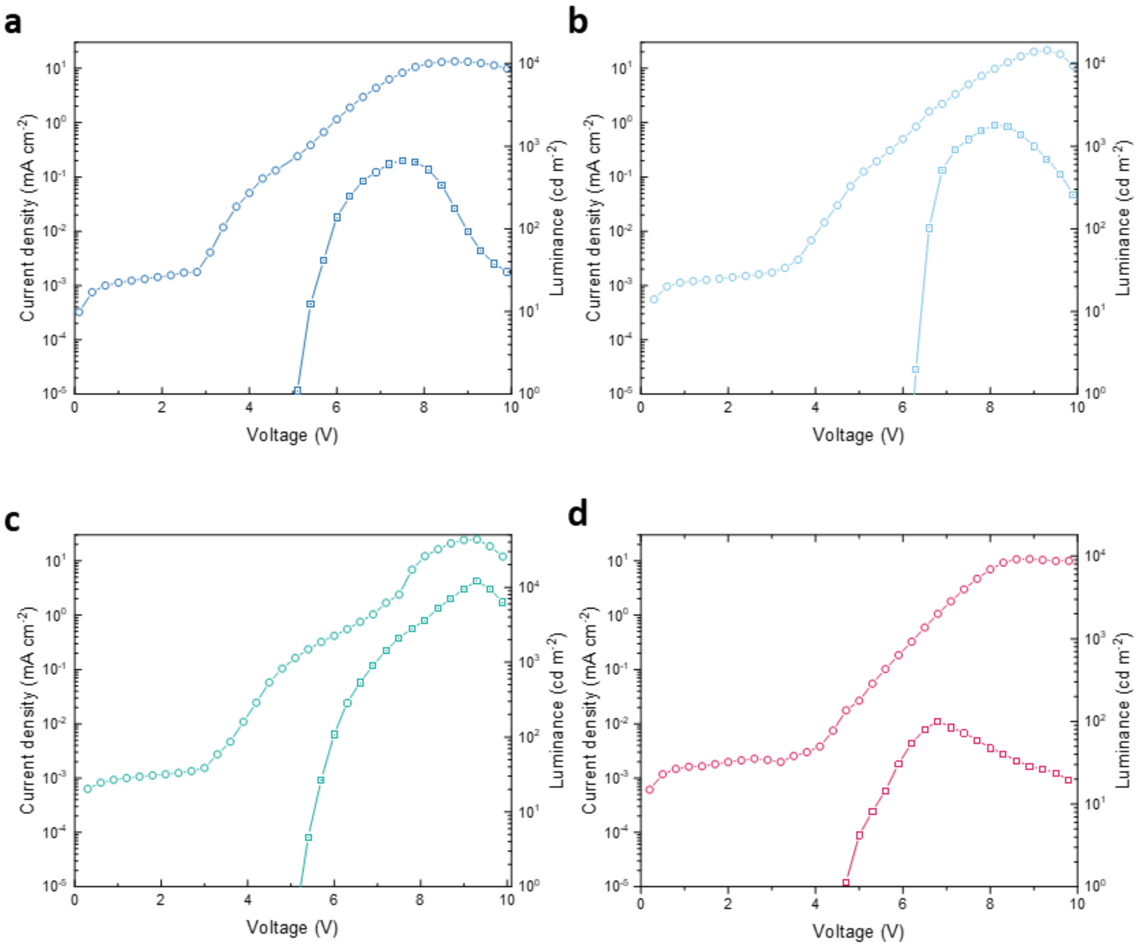

Supplementary Figure 16. The champion luminance of PeQWs-based LED device. Current density (*J*)-voltage (*V*) and luminance (*L*)-voltage (*V*) curves of **a**, 6-4 Cl-Br PeQWs. **b**, 4-6 Cl-Br PeQWs. **c**, $CsPbBr_3$ QWs. **d**, I-Br PeQWs.

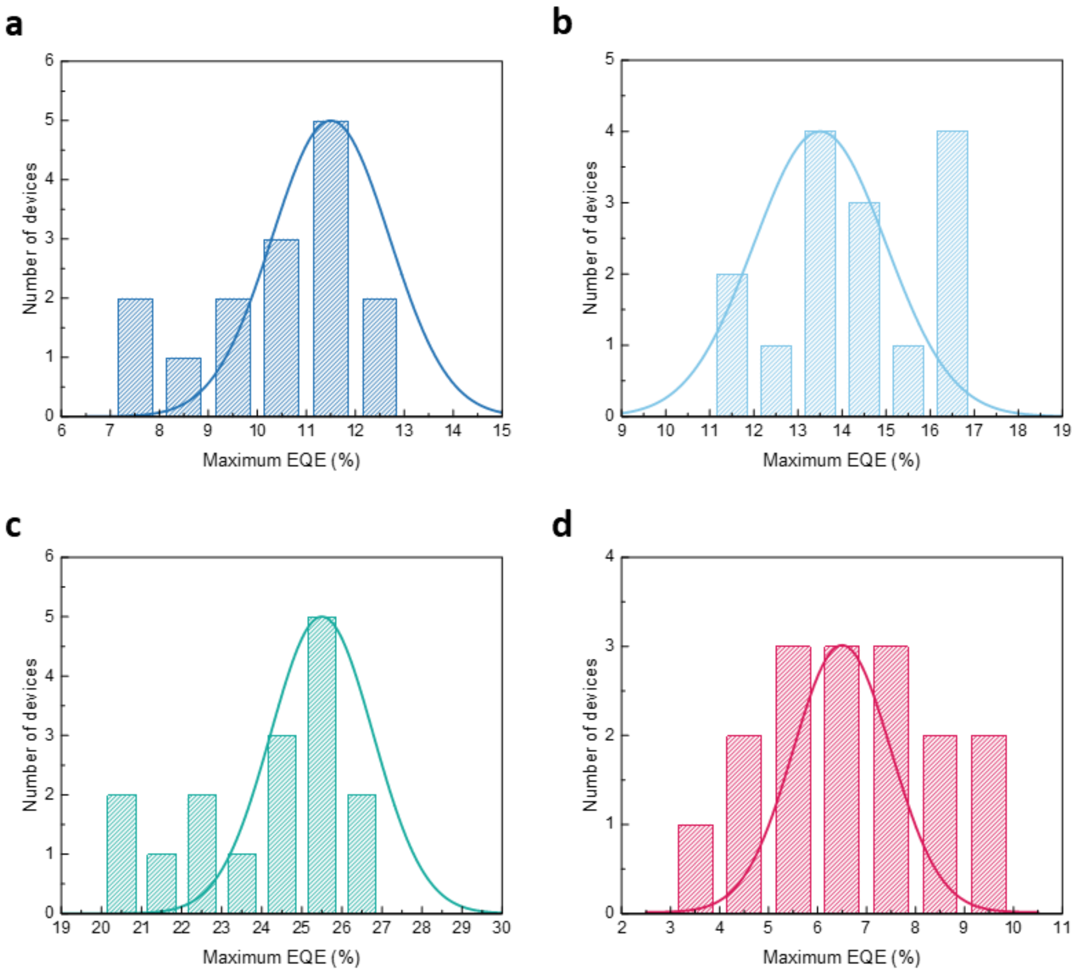

Supplementary Figure 17. Statistical data for device performance. Histograms of maximum EQEs for **a**, 6-4 Cl-Br PeQWs, **b**, 4-6 Cl-Br PeQWs, **c**, $CsPbBr_3$ QWs and **d**, I-Br PeQWs based LED devices.

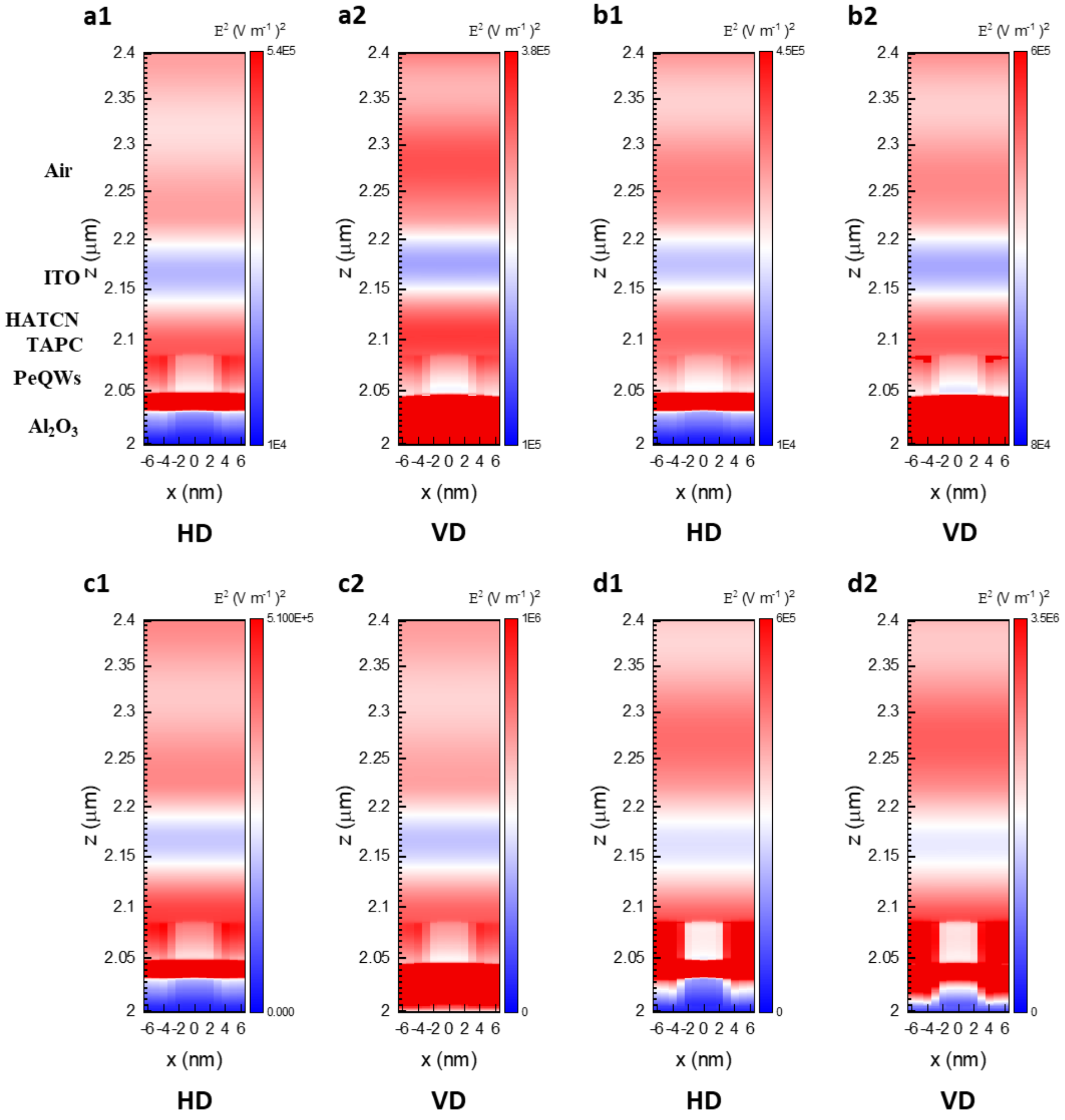

Supplementary Figure 18. Optical simulation. Cross-sectional E^2 intensity profiles of horizontal dipole (HD) and vertical dipole (VD) for **a1-a2**, 6-4 Cl-Br PeQWs, **b1-b2**, 4-6 Cl-Br PeQWs, **c1-c2**, $CsPbBr_3$ QWs and **d1-d2**, I-Br PeQWs based LED deivces.

Perovskite QWs	Dipole polarization	Out-coupling efficiency (%)	Average out-coupling efficiency (%)
6-4 Cl-Br QWs	HD	87.15	77.67
	VD	58.10	
4-6 Cl-Br QWs	HD	87.22	78.75
	VD	61.82	
Pure Br QWs	HD	86.89	79.52
	VD	64.80	
I-Br QWs	HD	83.91	82.06
	VD	78.46	

Supplementary Table 1. Summary of simulated outcoupling efficiency for PeQWs based LED devices.

The optical model we used is similar to our previous report and it can be found in Figure S4[1]. According to the supplementary note 2 of our previous report[2], the effective refractive index of perovskite QWs and PAM template is 1.79 (478 nm), 1.80 (491 nm), 1.81 (511 nm) and 1.92 (630 nm) for 6-4 Cl-Br PeQWs, 4-6 Cl-Br PeQWs, $CsPbBr_3$ QWs and I-Br PeQWs, respectively. Compared with perovskite itself, embedding perovskite in our PAM template reduces the effective refractive index of the emitting layer, greatly relieving the non-desired light-trapping effect usually defined by $1/n^2$ where n is the index of the emitting layer.

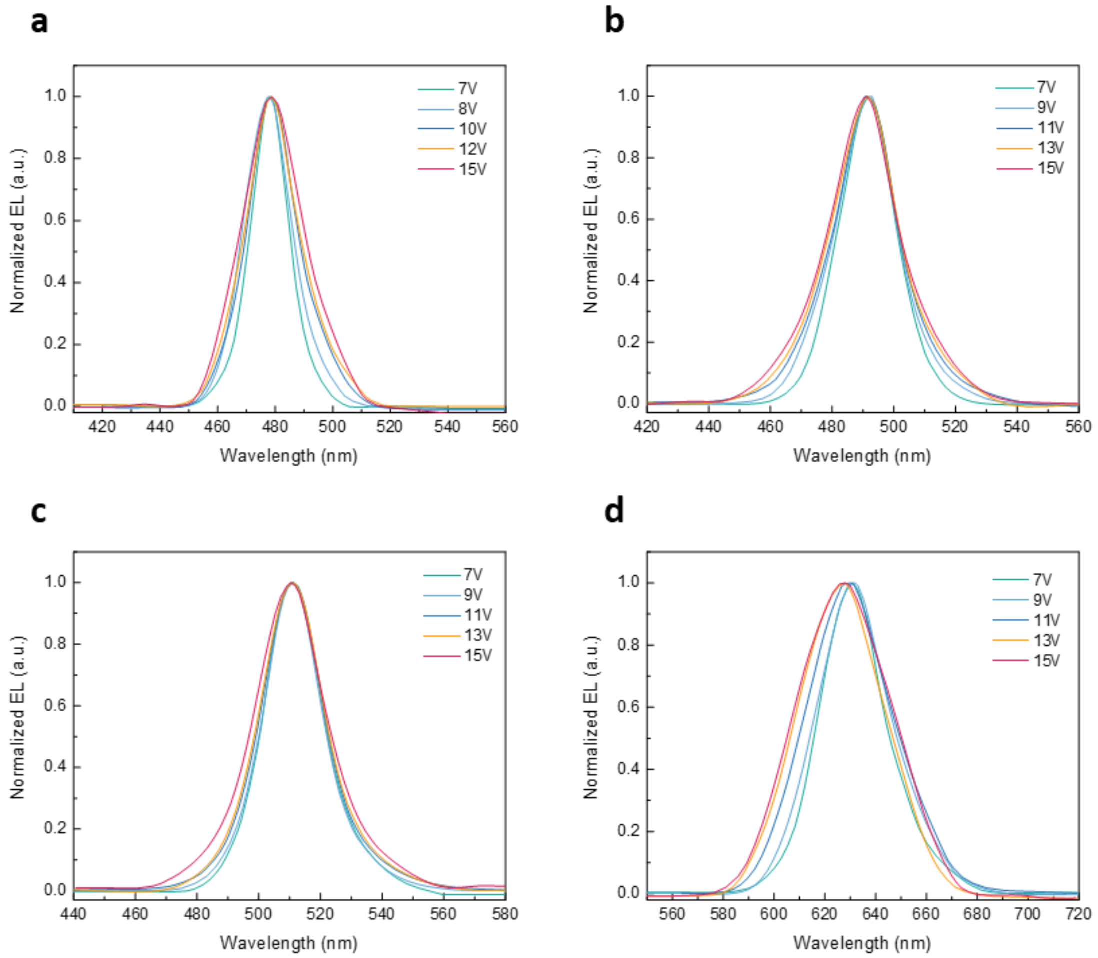

Supplementary Figure 19. Stability of the electroluminescence spectra of PeQWs-based LEDs. Electroluminescence spectra as a function of electric bias (>10 V) for **a**, 6-4 Cl-Br PeQWs, **b**, 4-6 Cl-Br PeQWs, **c**, $CsPbBr_3$ QWs and **d**, I-Br PeQWs based LED deivces.

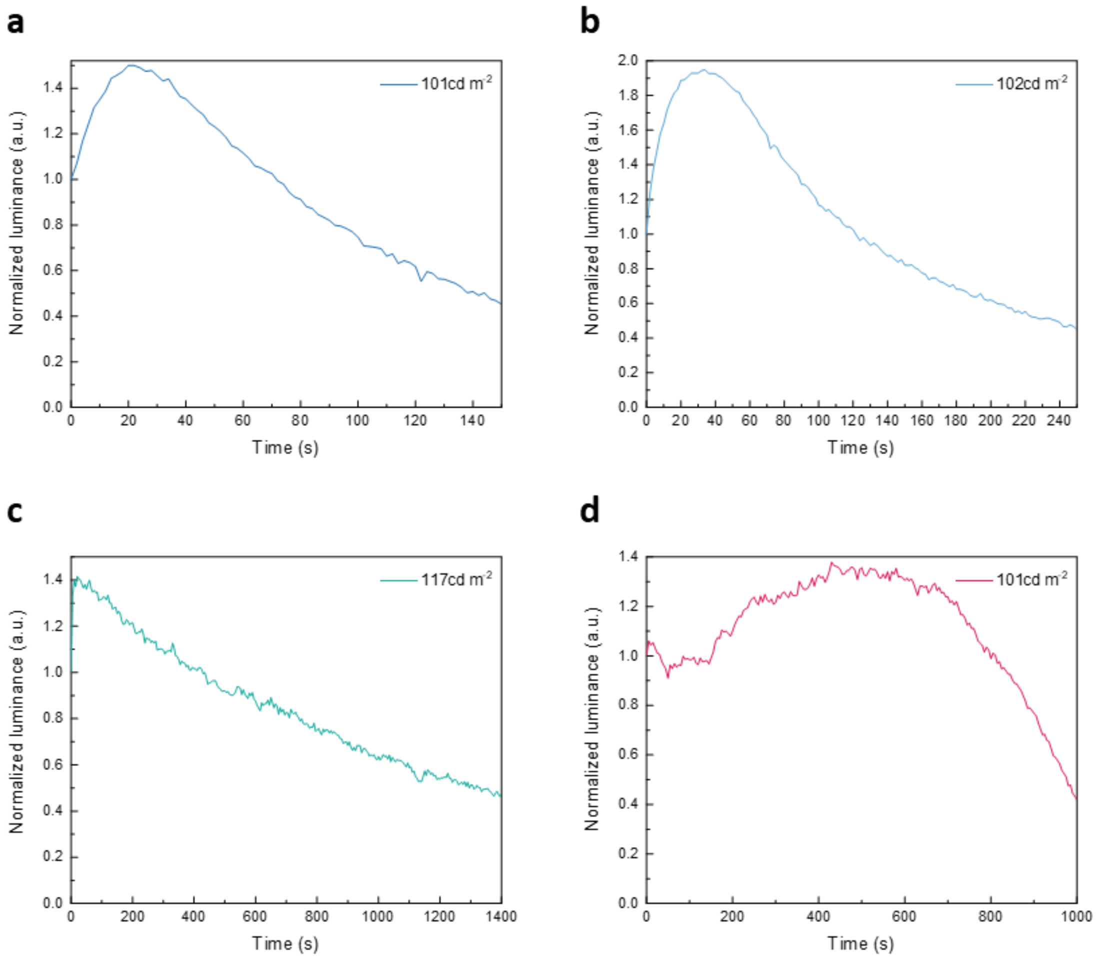

Supplementary Figure 20. Operation stability of PeQWs-based LEDs. T_{50} lifetime of **a**, 6-4 Cl-Br PeQWs, **b**, 4-6 Cl-Br PeQWs, **c**, $CsPbBr_3$ QWs and **d**, I-Br PeQWs based deivces under different luminance.

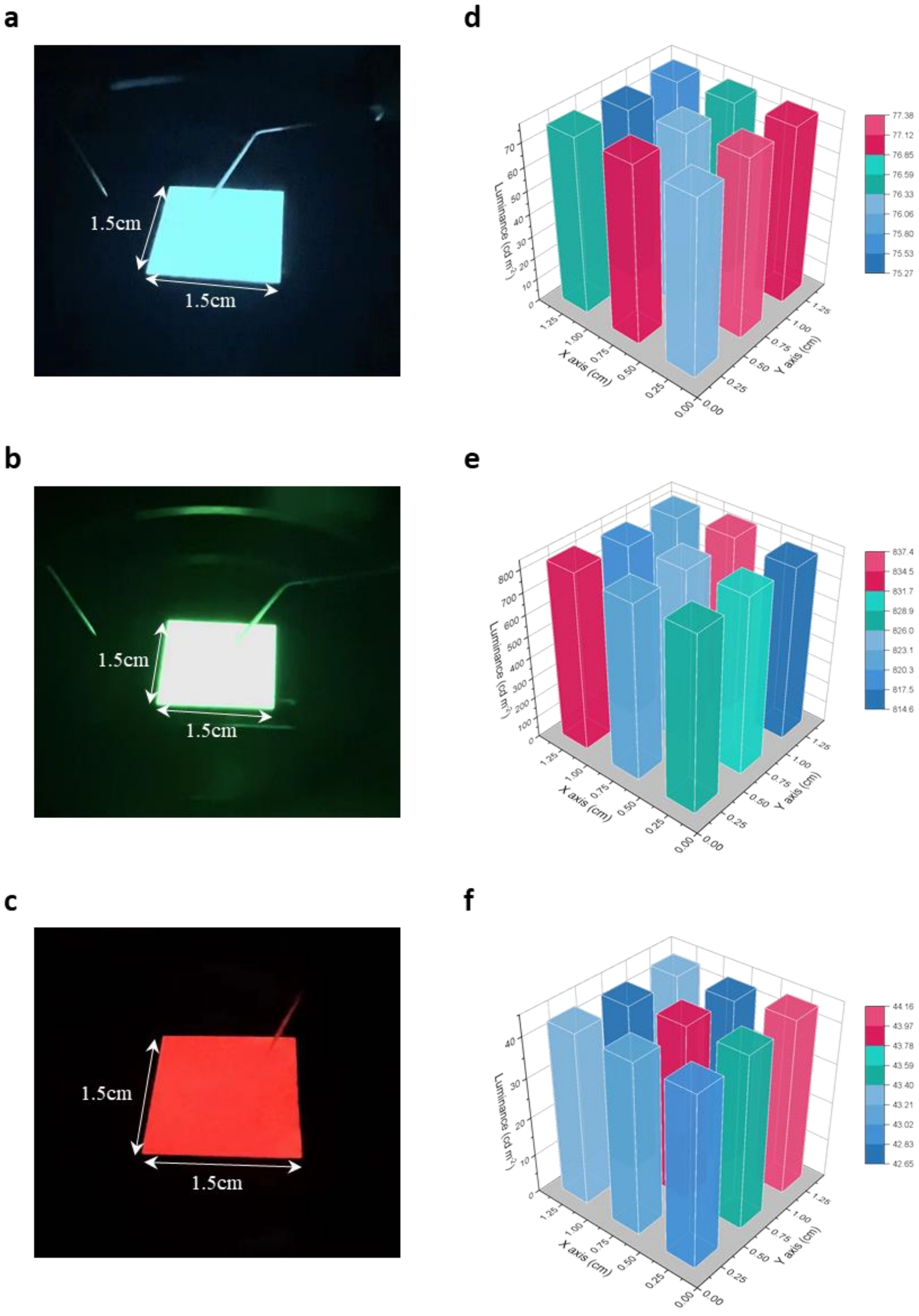

Supplementary Figure 21. 1.5×1.5 cm^2 PeQWs-based LED device. a-c, The photo image of large-area PeQWs-based LEDs with an emission area of 1.5×1.5 cm^2. **d-f,** The corresponding luminance distribution under working condition. Luminance is presented by a 3×3-pixel 3D column chart.

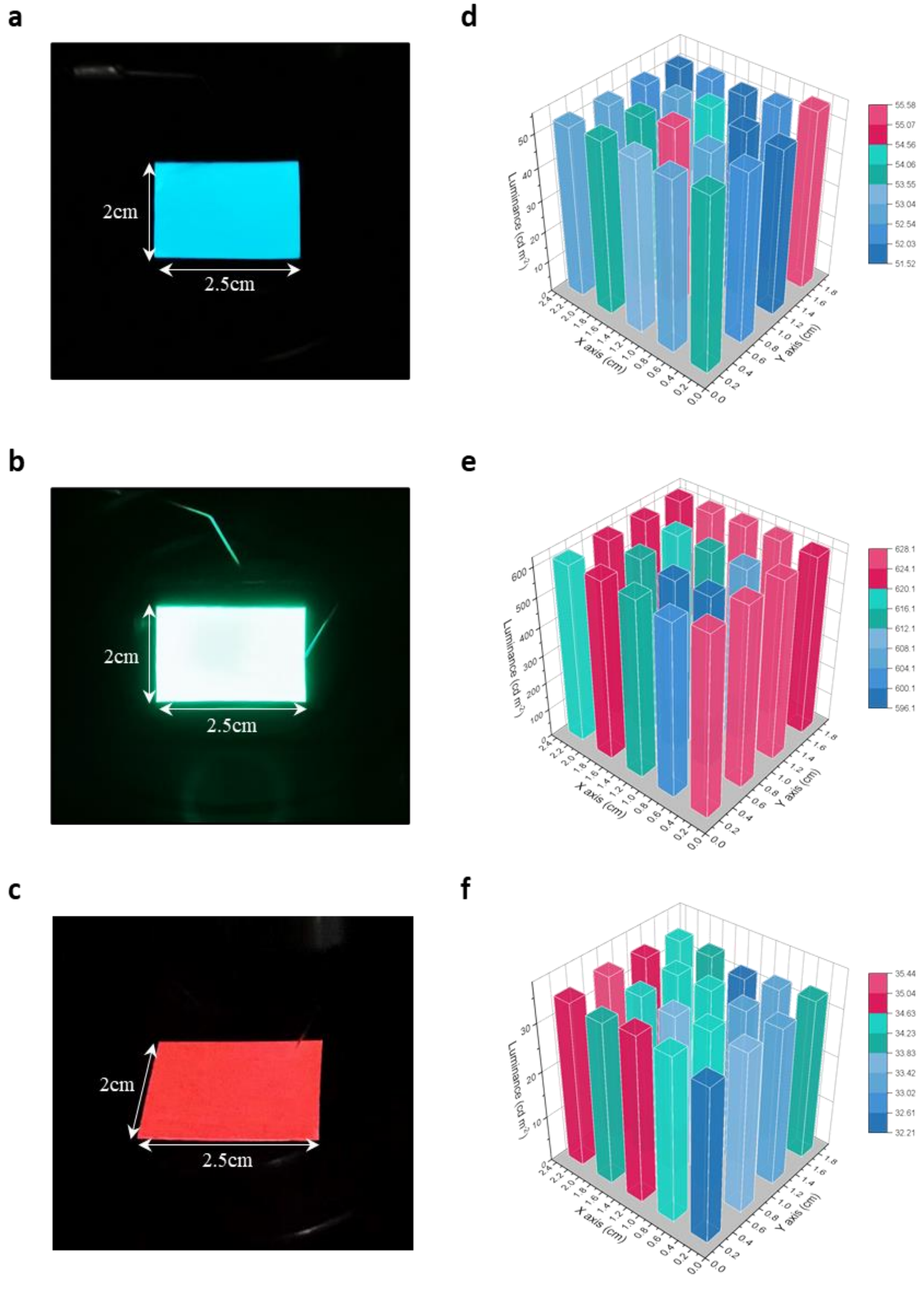

Supplementary Figure 22. 2×2.5 cm^2 PeQWs-based LED device. a-c, The photo image of large-area PeQWs-based LEDs with an emission area of 2×2.5 cm^2. **d-f,** The corresponding luminance distribution under working condition. Luminance is presented by a 4×5-pixel 3D column chart.

Supplementary Table 2. Time-resolved photoluminescence lifetimes of PeQWs with and without TAPC.

	τ_{avr} (ns)	τ_1 (ns)	τ_2 (ns)	χ^2
6-4 Cl-Br QWs	5.45	1.36 (32.23%)	7.39 (67.77%)	3.281
4-6 Cl-Br QWs	6.87	1.78 (30.99%)	9.15 (69.01%)	2.487
Pure Br QWs	7.40	1.90 (26.94%)	9.43 (73.06%)	2.282
I-Br QWs	12.94	1.20 (2.94%)	13.30 (97.06%)	4.313
6-4 Cl-Br QWs coated with TAPC and TBTB	4.36	1.63 (50.48%)	7.38 (47.52%)	3.285
4-6 Cl-Br QWs coated with TAPC and TBTB	4.78	1.87 (48.30%)	7.50 (51.40%)	2.847
Pure Br QWs coated with TAPC and TBTB	5.45	1.93 (40.55%)	7.85 (59.45%)	3.755
I-Br QWs coated with TAPC and TBTB	9.22	1.62 (14.38%)	10.50 (85.62%)	3.867

Supplementary Table 3. Performance of reported top-emitting perovskite LEDs

Perovskites	EQE_{max} (%)	CIE (x, y)	L_{max} $(cd\ m^{-2})$	λ_{EL} (nm)	Reference
6-4 Cl-Br QWs	12.41	(0.11, 0.13)	670	478	This work
4-6 Cl-Br QWs	16.49	(0.08, 0.33)	1788	491	
Pure Br QWs	26.09	(0.06, 0.71)	12147	512	
I-Br QWs	9.97	(0.70, 0.30)	101	630	
QDs: $CsPbI_3$	12.6	(0.72, 0.28)	10171	682	**Ref.** 3
RDP: $PEABr{:}CsPbBr_3$	13.6	(NA	~11000	512	**Ref.** 4
QDs: $Zr^{2+}{:}CsPbI_3$	13.7	(0.71, 0.28)	14725	686	**Ref.** 5
3D perovskites: $FAPbI_3$	20.2	NA	114.9 $mW\ sr^{-1}\ m^{-2}$	~800	**Ref.** 6

EQE_{max}, maximum external quantum efficiency; CIE, Commission Internationale de l´Eclairage; L_{max}, maximum luminance; R_{max}, maximum radiance; λ_{EL}, EL wavelength; RDP, reduced dimensional perovskite; QDs, quantum dots.

Supplementary Table 4. Performance of reported all-inorganic perovskite LEDs

Perovskites	EQE_{max} (%)	CIE (x, y)	L_{max} ($cd\ m^{-2}$)	λ_{EL} (nm)	Reference
6-4 Cl-Br QWs	12.41	(0.11, 0.13)	670	478	This work
4-6 Cl-Br QWs	16.49	(0.08, 0.33)	1788	491	
Pure Br QWs	26.09	(0.06, 0.71)	12147	512	
I-Br QWs	9.97	(0.70, 0.30)	101	630	
QDs: K^+:$CsPb(Br/Cl)_3$	1.96	(0.11, 0.12)	212.9	476	**Ref.** 7
3D perovskites: Rb^+:$CsPb(Br/Cl)_3$	11.0	(0.107, 0.115)	2180	477	**Ref.** 8
QDs: $CsPbBr_3$	12.3	(NA, 0.13)	~400	479	**Ref.** 9
RDP: YCl_3:$CsPbBr_3$	13.5	(0.07, 0.25)	2224	488	**Ref.** 10
QDs: $CsPb(Br/Cl)_3$	1.9	NA	35	490	**Ref.** 11
RDP: $CsPbBr_3$	13.8	(0.06, 0.36).	2825	496	**Ref.** 12
QDs: $CsPbBr_3$	22.0	NA	~1000	505	**Ref.** 9
3D perovskites: $CsPbBr_3$	16.45	NA	112824	522	**Ref.** 13
QDs: K^+:$CsPbI_3$	23	(0.69, 0.30)	~1000	640	**Ref.** 14

EQE_{max}, maximum external quantum efficiency; CIE, Commission Internationale de l´Eclairage; L_{max}, maximum luminance; λ_{EL}, EL wavelength; RDP, reduced dimensional perovskite; QDs, quantum dots.

Supplementary References

1 Zhang, Q. *et al.* Three-Dimensional Perovskite Nanophotonic Wire Array-Based Light-Emitting Diodes with Significantly Improved Efficiency and Stability. *ACS Nano* **14**, 1577-1585 (2020).

2 Zhang, Q. *et al.* Efficient metal halide perovskite light-emitting diodes with significantly improved light extraction on nanophotonic substrates. *Nature Communications* **10**, 727 (2019).

3 Lu, M. *et al.* Surface ligand engineering-assisted CsPbI3 quantum dots enable bright and efficient red light-emitting diodes with a top-emitting structure. *Chemical Engineering Journal* **404**, 126563 (2021).

4 Cai, L. *et al.* High-Efficiency Top-Emitting Green Perovskite Light Emitting Diode with Quasi Lambertian Emission. *Advanced Optical Materials* **n/a**, 2101137.

5 Lu, M. *et al.* Bright CsPbI3 Perovskite Quantum Dot Light-Emitting Diodes with Top-Emitting Structure and a Low Efficiency Roll-Off Realized by Applying Zirconium Acetylacetonate Surface Modification. *Nano Letters* **20**, 2829-2836 (2020).

6 Miao, Y. *et al.* Microcavity top-emission perovskite light-emitting diodes. *Light: Science & Applications* **9**, 89 (2020).

7 Yang, F. *et al.* Efficient and Spectrally Stable Blue Perovskite Light-Emitting Diodes Based on Potassium Passivated Nanocrystals. *Advanced Functional Materials* **30**, 1908760 (2020).

8 Karlsson, M. *et al.* Mixed halide perovskites for spectrally stable and high-efficiency blue light-emitting diodes. *Nature Communications* **12**, 361 (2021).

9 Dong, Y. *et al.* Bipolar-shell resurfacing for blue LEDs based on strongly confined perovskite quantum dots. *Nature Nanotechnology* **15**, 668-674 (2020).

10 Liu, Y. *et al.* Water-Soluble Conjugated Polyelectrolyte Hole Transporting Layer for Efficient Sky-Blue Perovskite Light-Emitting Diodes. *Small* **17**, 2101477 (2021).

11 Pan, J. *et al.* Highly Efficient Perovskite-Quantum-Dot Light-Emitting Diodes by Surface Engineering. *Advanced Materials* **28**, 8718-8725 (2016).

12 Zhu, Z. *et al.* Highly Efficient Sky-Blue Perovskite Light-Emitting Diode Via Suppressing Nonradiative Energy Loss. *Chemistry of Materials* **33**, 4154-4162 (2021).

13 Feng, W. *et al.* Efficient all-inorganic perovskite light-emitting diodes enabled by manipulating the crystal orientation. *Journal of Materials Chemistry A* **9**, 11064-11072 (2021).

14 Wang, Y.-K. *et al.* All-Inorganic Quantum-Dot LEDs Based on a Phase-Stabilized α-CsPbI3 Perovskite. *Angewandte Chemie International Edition* **60**, 16164-16170 (2021).
