# Supplementary material for: High-efficiency, flexible and large-area red/green/blue all-inorganic metal halide perovskite quantum wires-based light-emitting diodes
Source: Nat Commun. 2023 Aug 1;14:4611. doi: 10.1038/s41467-023-40150-y (PMC10393990; doi:10.1038/s41467-023-40150-y)
Supplement: Supplementary file 2 — Peer Review File [file 41467_2023_40150_MOESM2_ESM.pdf]

nature portfolio

Peer Review File

High-Efficiency, Flexible and Large-area Red/Green/Blue All-Inorganic Metal Halide Perovskite Quantum Wires-Based Light-Emitting Diodes

Open Access This file is licensed under a Creative Commons Attribution 4.0 International License, which permits use, sharing, adaptation, distribution and reproduction in any medium or format, as long as you give appropriate credit to the original author(s) and the source, provide a link to the Creative Commons license, and indicate if changes were made. In the cases where the authors are anonymous, such as is the case for the reports of anonymous peer reviewers, author attribution should be to 'Anonymous Referee' followed by a clear attribution to the source work. The images or other third party material in this file are included in the article's Creative Commons license, unless indicated otherwise in a credit line to the material. If material is not included in the article's Creative Commons license and your intended use is not permitted by statutory regulation or exceeds the permitted use, you will need to obtain permission directly from the copyright holder. To view a copy of this license, visit http://creativecommons.org/licenses/by/4.0/.

Editorial Note: This manuscript has been previously reviewed at another journal that is not operating a transparent peer review scheme. This document only contains reviewer comments and rebuttal letters for versions considered at *Nature Communications* .

REVIEWER COMMENTS

Reviewer #2 (Remarks to the Author):

This revised manuscript should be suitable for Nature Communications. Here are the remaining concerns.

1) I must be convinced that the Cl-Br anions are evenly distributed, as it is one of the central claims in this manuscript. It is clear that the EL emission is not symmetrical from Fig. S5. If it arises from a high density of trap states, how is it possible to show the high PLQYs and decent device performance? As I said, it is not reasonable to draw a conclusion simply by TA photobleaching. Even for the samples with severe heterogeneity, one may see a single but broadened photobleaching band like the work of Feng Gao's group. In the case without a comparison, I do not believe the conclusion is solid. I recommend that the authors do additional tests like Urbach energy or hyperspectral imaging to evaluate the heterogeneity.

2) What makes the UV-Vis absorption of I-Br QWs so weird?

3) I still cannot be convinced that the perovskites are quantum-confined. I suggest the authors test fluence-dependent PLQYs or PL decays to investigate recombination kinetics.

Reviewer #2 (Remarks to the Author):

This revised manuscript should be suitable for Nature Communications. Here are the remaining concerns.
Response: We sincerely thank the reviewer for the positive recognition.

1) I must be convinced that the Cl-Br anions are evenly distributed, as it is one of the central claims in this manuscript. It is clear that the EL emission is not symmetrical from Fig. S15. If it arises from a high density of trap states, how is it possible to show the high PLQYs and decent device performance? As I said, it is not reasonable to draw a conclusion simply by TA photobleaching. Even for the samples with severe heterogeneity, one may see a single but broadened photobleaching band like the work of Feng Gao's group. In the case without a comparison, I do not believe the conclusion is solid. I recommend that the authors do additional tests like Urbach energy or hyperspectral imaging to evaluate the heterogeneity.
Response: We thank the reviewer for the valuable question and suggestion. In the manuscript, the statement is "According to the TA spectra in Supplementary Fig. 7, a single relatively narrow GSB peak can be found, indicating that no energy transfer is observed and PeQW is of high compositionally homogenous. Here, the term "compositional homogeneity" indicates the halide anions are uniformly distributed in mixed halide perovskite. It has been reported that a high compositional homogeneity can significantly improve the phase stability, thus suppressing the halide segregation.". We would like to emphasize that our PeQWs have a relatively high compositional homogeneity, but it doesn't mean that the Cl-Br anions are completely evenly distributed. We have revised the statement to avoid any misunderstanding and marked it in red. If an electric bias is applied to device, perovskite will experience ion migration and lattice distortion, leading to a broadened and asymmetric EL emission. In Figure. S15, we applied >7V bias which is quite high for mixed-halide perovskite. In the same bias, the EL emission changes dramatically in the work of Feng Gao's group. However, the emission peak position remains almost unchanged despite a broadening EL emission for our PeQWs-based devices.
To analyze the trap density of our PeQWs, we also performed Space charge-limited current (SCLC) measurement using a hole-only device structure (shown in Figure S1). We calculated the total trap state density inside the PeQWs by the equation as below:

$$n_t = \frac{2\varepsilon\varepsilon_0 V_{TFL}}{eL^2}$$

where n_t is the trap state density, V_{TFL} is the trap-filled limit voltage, L is the thickness of the perovskites, e is the elementary charge, ε_0 and ε are the vacuum permittivity and relative permittivity, respectively. The calculated lowest trap density is $1.77 \times 10^{16}\ cm^{-3}$ which is comparable to the value reported in an earlier work (Nature 611, 688–694).

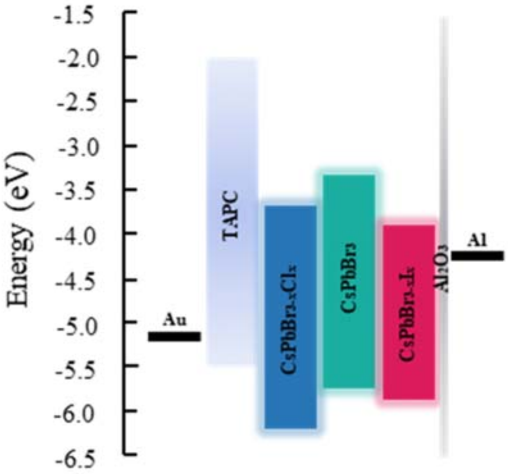

Figure S1. Device structure of hole-only devices

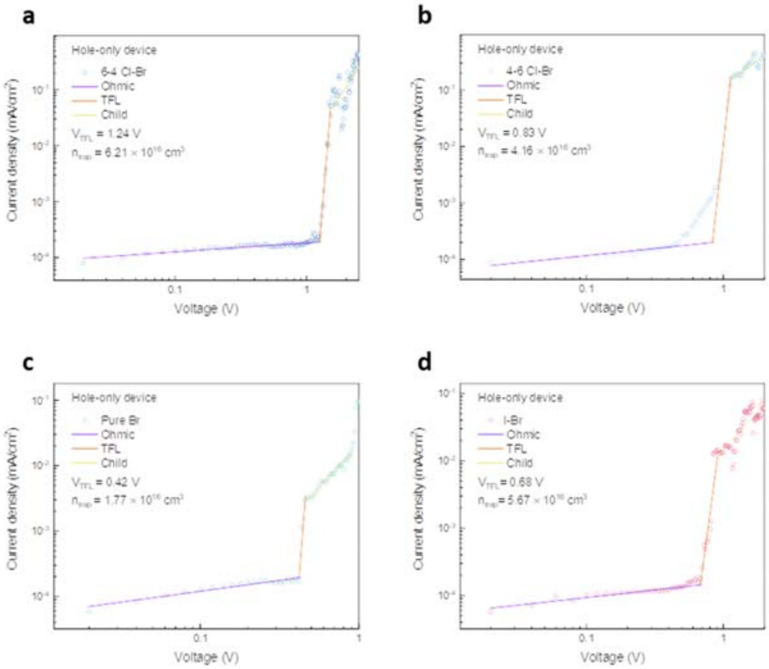

Figure S2. Space charge-limited current (SCLC) measurement for **a**, 6-4 Cl-Br PeQWs. **b**, 4-6 Cl-Br PeQWs. **c**, $CsPbBr_3$ QWs. **d**, I-Br PeQWs.

We extracted the absorption coefficient spectrum from the absorbance spectrum which are shown below. In this way, the Urbach energy can be calculated as follow:

$$\alpha = \alpha_0 e^{\frac{h\nu}{E_u}}$$

where α is the absorption coefficient and $h\nu$ is the photon energy. The Urbach energy was found to be 23.1 meV, 24.1 meV, 31.5 meV and 89.1 meV for 6-4 Cl-Br, 4-6 Cl-Br, $CsPbBr_3$ and I-Br PeQWs, respectively. The Urbach energy is a parameter used to quantify energetic disorder in the band edges of a semiconductor. The low Urbach energy indicates a low

electronic disorder in the band edges for our PeQWs (ACS Appl. Mater. Interfaces 2022, 14, 6, 7796–7804). The PL full width of half maximum (FWHM) is 19 nm, 19 nm, 18 nm and 30 nm for 6-4 Cl-Br, 4-6 Cl-Br, $CsPbBr_3$ and I-Br PeQWs, respectively. The narrow emission linewidth also suggests that the Cl and Br anions are not completely separated into different domains. Therefore, we believe that conclusion can be drawn that our PeQWs have a relatively high compositional homogeneity based on the evidence presented.

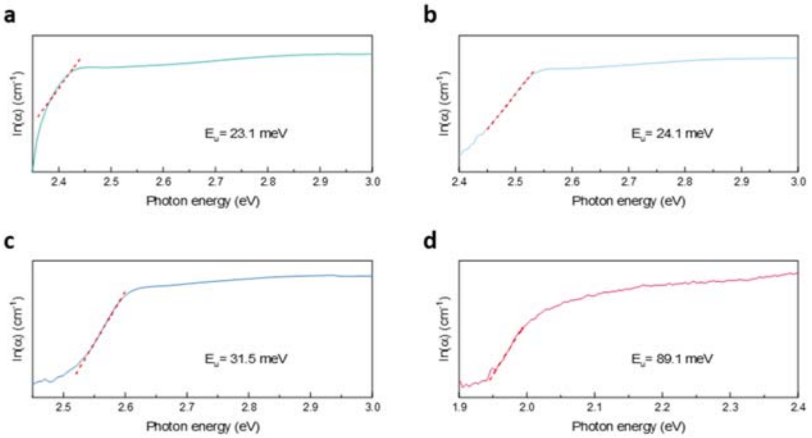

Figure S3. Absorption coefficient spectra for **a**, 6-4 Cl-Br PeQWs. **b**, 4-6 Cl-Br PeQWs. **c**, $CsPbBr_3$ QWs. **d**, I-Br PeQWs.

2) What makes the UV-Vis absorption of I-Br QWs so weird?

Response: We thank the reviewer for raising this comment. We fabricated new samples and measured the absorbance of I-Br PeQWs again. The new result is shown in the figure below, and we believe it is quite normal as it is similar to the result from the earlier work (Nature 612, 679–684). We attribute the weird UV-Vis absorption of old sample to either poor quality after fabrication or material degradation after being taken out of the glovebox for measurement.

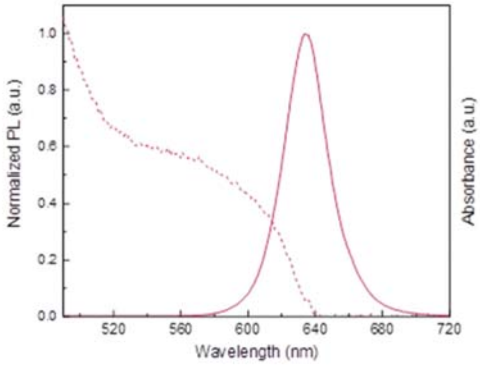

Figure S4. Photoluminescence and absorbance spectrum of I-Br PeQWs.

3) I still cannot be convinced that the perovskites are quantum-confined. I suggest the authors

test fluence-dependent PLQYs or PL decays to investigate recombination kinetics.

Response: We thank the reviewer for the valuable advice. Unfortunately, we are unable to measure fluence-dependent PLQY or PL decays at present because our equipment is not equipped with an intensity-tunable laser diode. However, we did carry out temperature-dependent PL to evaluate the exciton binding energy of our PeQWs. The temperature-dependent PL spectra are shown in Figure S6. We obtain the estimate of exciton binding energy by the temperature dependence of integrated PL signal in terms of the aforementioned method (Nature 612, 679–684):

$$I(T) = \frac{I_0}{1 + Ae^{-\frac{E_b}{k_B T}}}$$

where I_0 is the integrated PL intensity extrapolated at 0 K, A is a constant, E_b is the exciton binding energy, and k_b is the Boltzmann constant. Note that the exciton binding energy of perovskite might be over-estimated due to the possibility of structural transition at low temperature. As a result, the exciton binding energy is approximately 271 meV, 207 meV, 161 meV and 106 meV for 6-4 Cl-Br, 4-6 Cl-Br, $CsPbBr_3$ and I-Br PeQWs, respectively. For 3D perovskite thin film, the exciton binding energy is usually less than 100 meV (Nature 612, 679–684, Nature 611, 688–694 and Sci Rep 6, 28618). The high exciton binding energy of our PeQWs indicates that our perovskite experiences a strong quantum confinement effect from the diameter reduction (Nature 612, 679–684 and Nature 611, 688–694). Additionally, the exciton Bohr diameter of $CsPbBr_3$ is 7 nm (Nature 612, 679–684) and the diameter of our PeQWs is around 6.4 nm (<7 nm). Thus, we are confident that our perovskite experiences confinement in two dimensions.

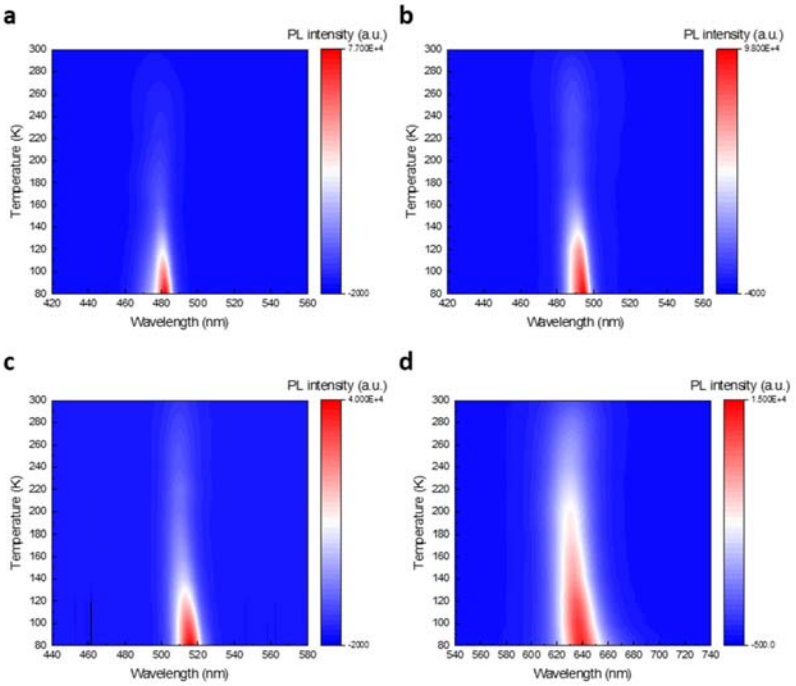

Figure S5. Temperature-dependent PL spectra of **a**, 6-4 Cl-Br PeQWs. **b**, 4-6 Cl-Br PeQWs.

c, $CsPbBr_3$ QWs. **d**, I-Br PeQWs.

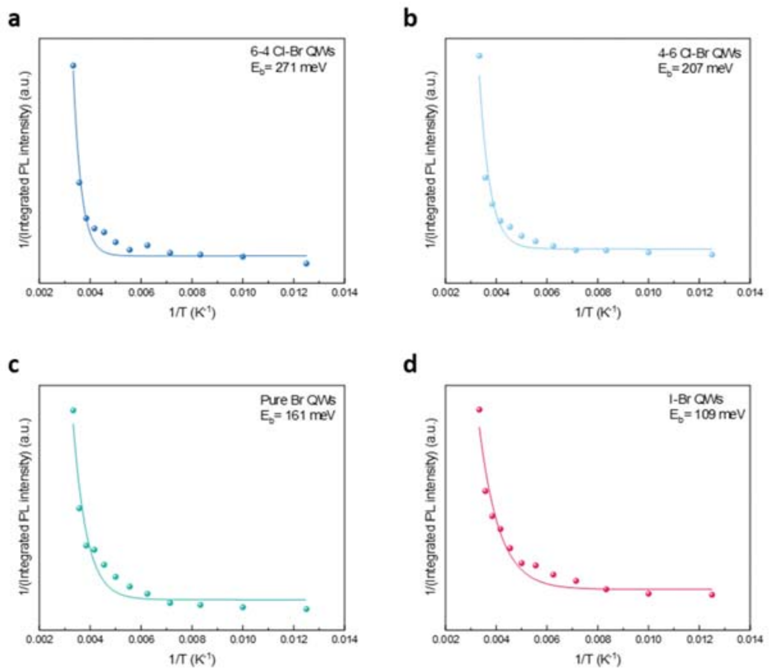

Figure S6. 1/(Integrated PL intensity) versus inverse temperature of **a**, 6-4 Cl-Br PeQWs. **b**, 4-6 Cl-Br PeQWs. **c**, $CsPbBr_3$ QWs. **d**, I-Br PeQWs.

REVIEWERS' COMMENTS

Reviewer #2 (Remarks to the Author):

This manuscript can be accepted without futher revision.
